# Supplementary material for: Immunofocusing design of a fusion glycoprotein monomer vaccine for respiratory syncytial virus
Source: Mol Ther. 2025 Jul 28;33(11):5401–14. doi: 10.1016/j.ymthe.2025.07.040 (PMC12628061; doi:10.1016/j.ymthe.2025.07.040)
Supplement: Document S1. Figures S1–S7 and Table S1 [file mmc1.pdf]

YMTHE, Volume *33*

Supplemental Information

Immunofocusing design of a fusion glycoprotein monomer vaccine for respiratory syncytial virus

Qianqian Li, Zhiming Li, Jiamin Chen, Qiuju He, Hongjian Xiao, Haiwei Li, Huan Li, Heng Zhang, Yaoyun Yang, Rong Bi, Zichen Li, Song Xiao, Yanwei Bi, Bingyan Liang, Luxia Huang, Mengyi Zhang, Jincheng Tong, Haoyue Long, Ru Li, Jinmei Duan, Zhihua Li, and Youchun Wang

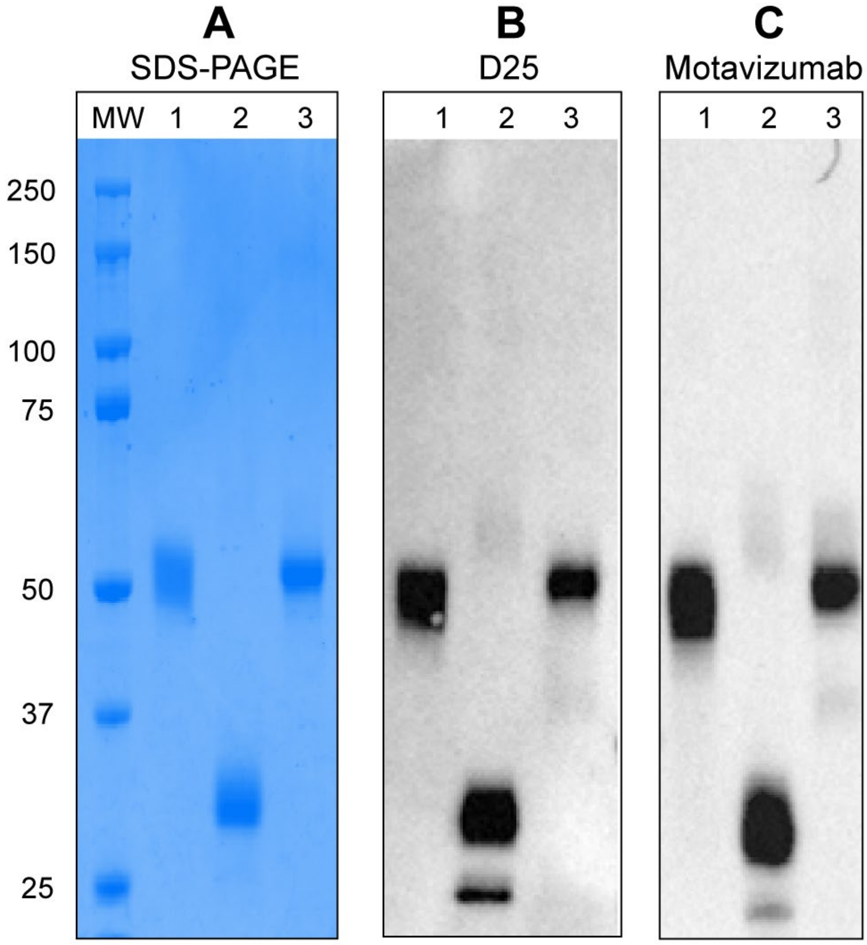

Figure S1. Characterization of RSV F immunogens, related to Figure 1. (A) Reduced SDS-PAGE analysis of purified RSV F immunogens. (B) Western blot analysis of purified RSV F immunogens using anti-RSV monoclonal antibody D25. (C) Western blot analysis of purified RSV F immunogens using anti-RSV monoclonal antibody Motavizumab. Lane1, Q74 trimer; lane2, Q74 monomer; lane3, DS-Cav1.

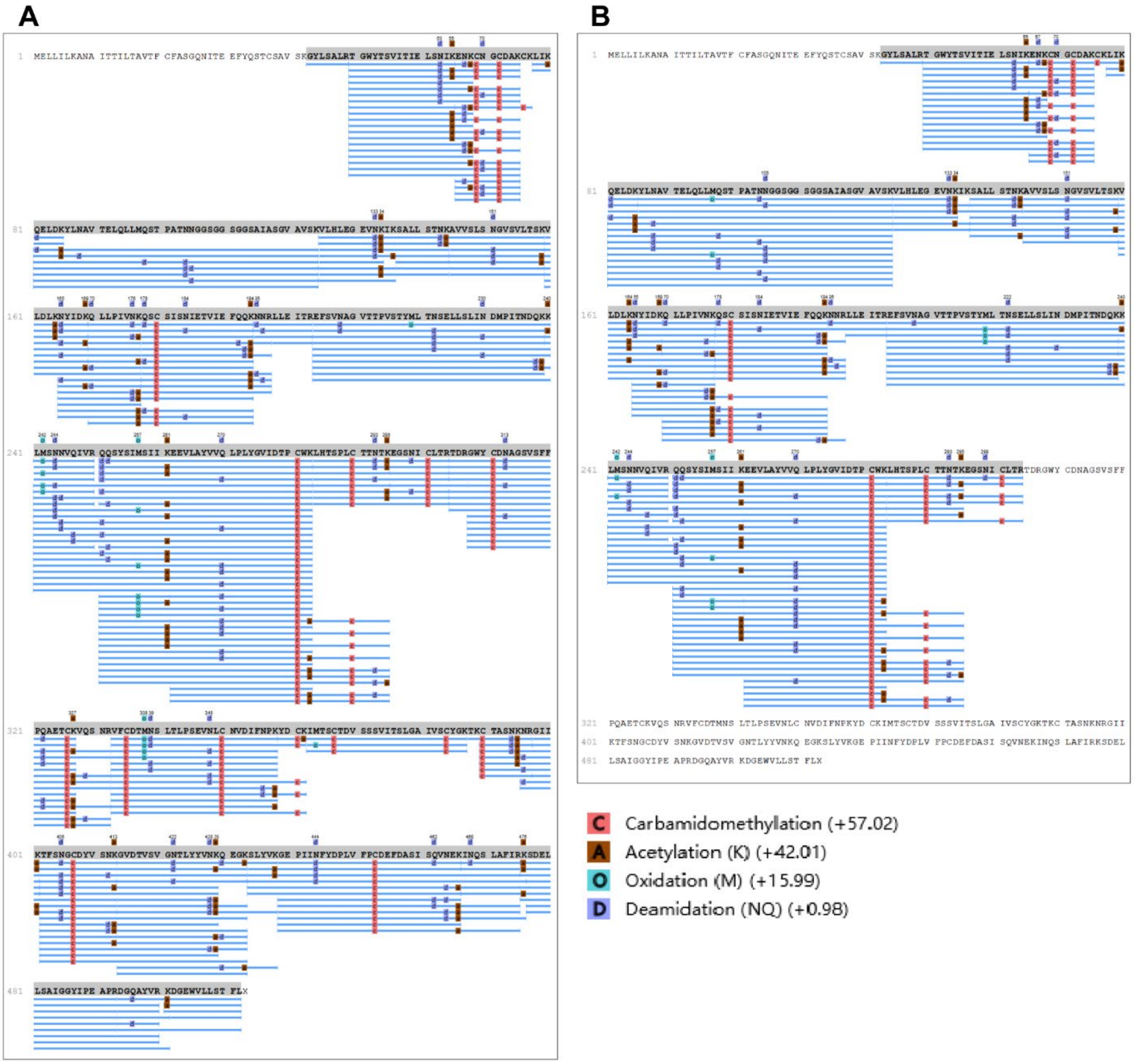

Figure S2. Peptide mapping analysis of RSV F immunogens, related to Figure 1. Sequence coverage of the Q74 trimer protein (A) and Q74 monomer protein (B) was calculated after identification of peptides detected in the peptide map. The amino acid sequence is shown with blue bars to indicate the identified regions. PTM was identified by searching the PEAKS database. The exact site of modification (carbamidomethylation, acetylation, oxidation and deamidation) were represented on the blue bars.

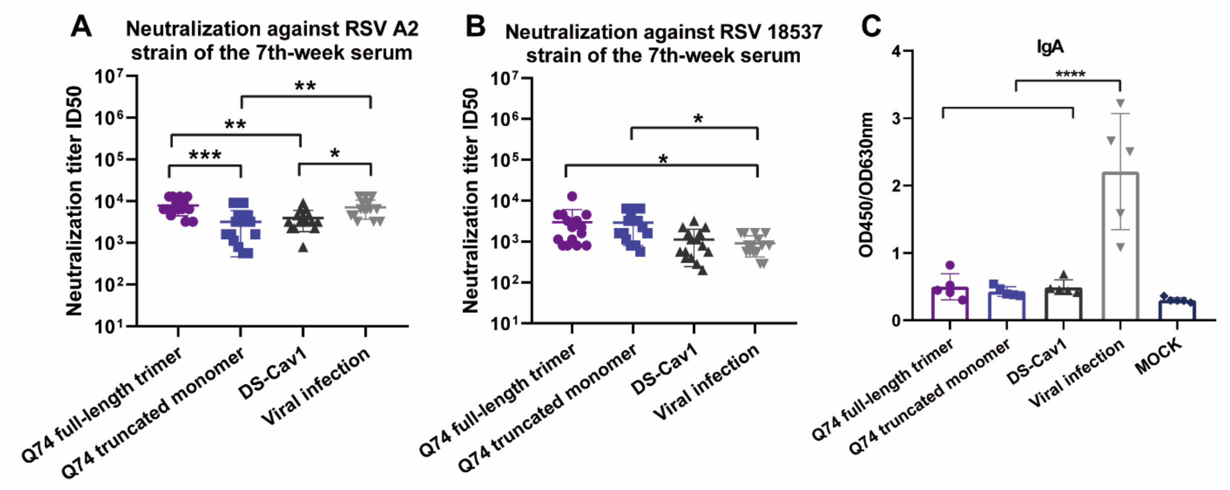

Figure S3. Evaluation of antibody response to RSV F immunogens in mice, related to Figure 4. Neutralizing antibody titers against RSV A2 (A) and B18537 (B) strains were assessed using a live virus neutralization assay in sera collected from mice (15 per group) at week 7 after immunization. Titers are reported as the 50% geometric mean titer (GMT) within each group as mean ± SD. GMTs were log10-transformed. (C) The fully immunized mice were euthanized 3 weeks after the 2ndboost immunization, and bronchoalveolar lavage fluid (BALF) was collected. Mucosal immunoreactivity in BALF samples was determined by assessing DS-CaV1-specific IgA. Statistical comparisons were performed using one-way analysis of variance (ANOVA) between different groups. * means $P < 0.05$; ** means $P < 0.01$; *** means $P < 0.001$; **** means $P < 0.0001$; ns means not significant.

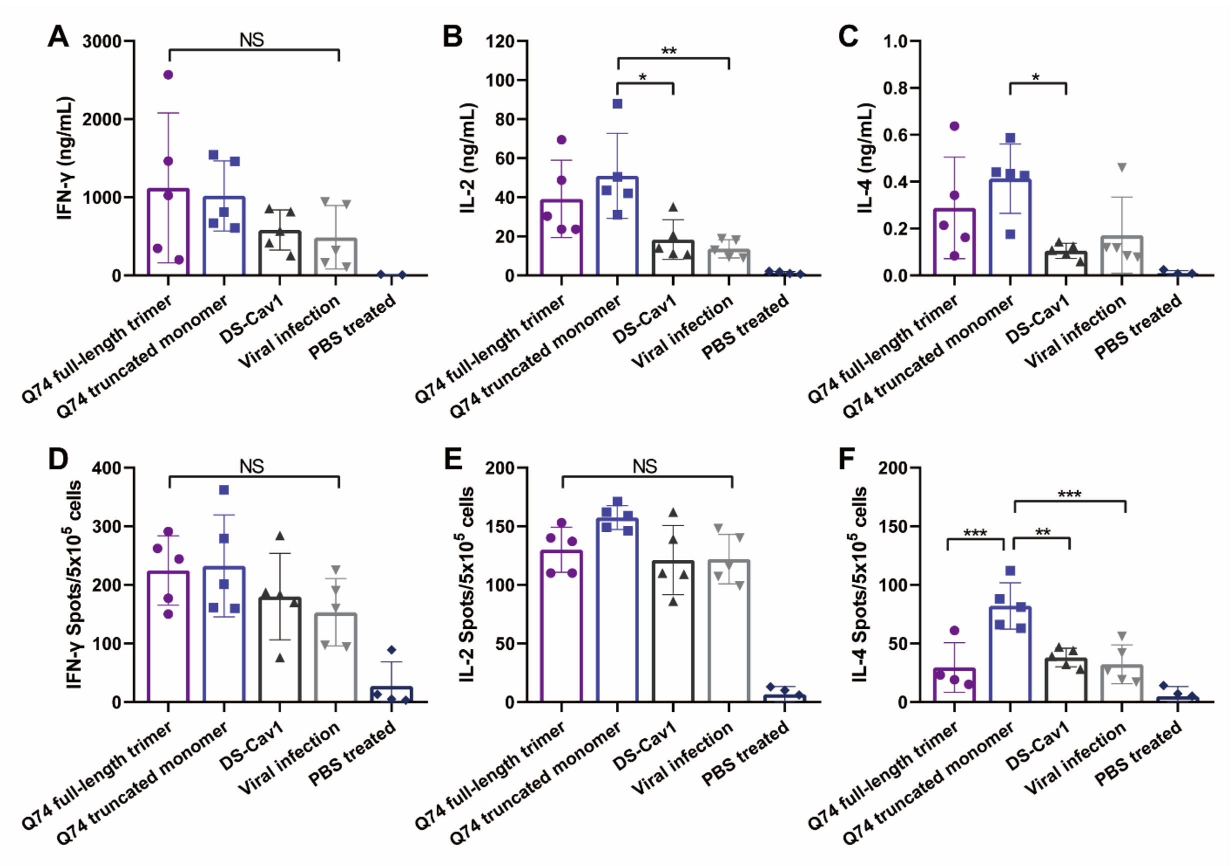

Figure S4. Evaluation of cellular immune response to RSV F immunogens in mice, related to Figure 4. The fully immunized mice were euthanized 3 weeks after the second booster immunization and splenic lymphocytes were collected. The levels of secreted IFN-γ (A), IL-2 (B) and IL-4 (C) in splenocytes after DS-CaV1 stimulation were assayed using ELISA. The numbers of IFN-γ- (D), IL-2- (E) and IL-4-secreting(F) splenocytes after DS-CaV1 stimulation were measured using Enzyme-linked immunospot assay (ELISpot). Statistical comparisons were performed using one-way analysis of variance (ANOVA) between different groups. * means $P < 0.05$; ** means $P < 0.01$; *** means $P < 0.001$; **** means $P < 0.0001$; ns means not significant.

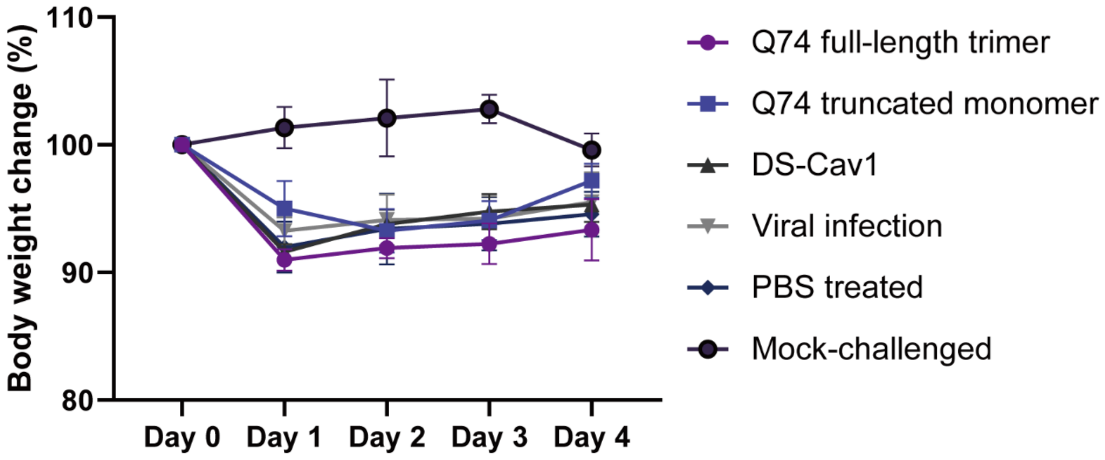

Figure S5. Body weight changes in mice following RSV infection, related to Figure 5. Daily body weight is shown as a percentage of the weight on day 0. PBS-treated: RSV-infected (positive control) group; Mock-challenged: Uninfected (negative control) group.

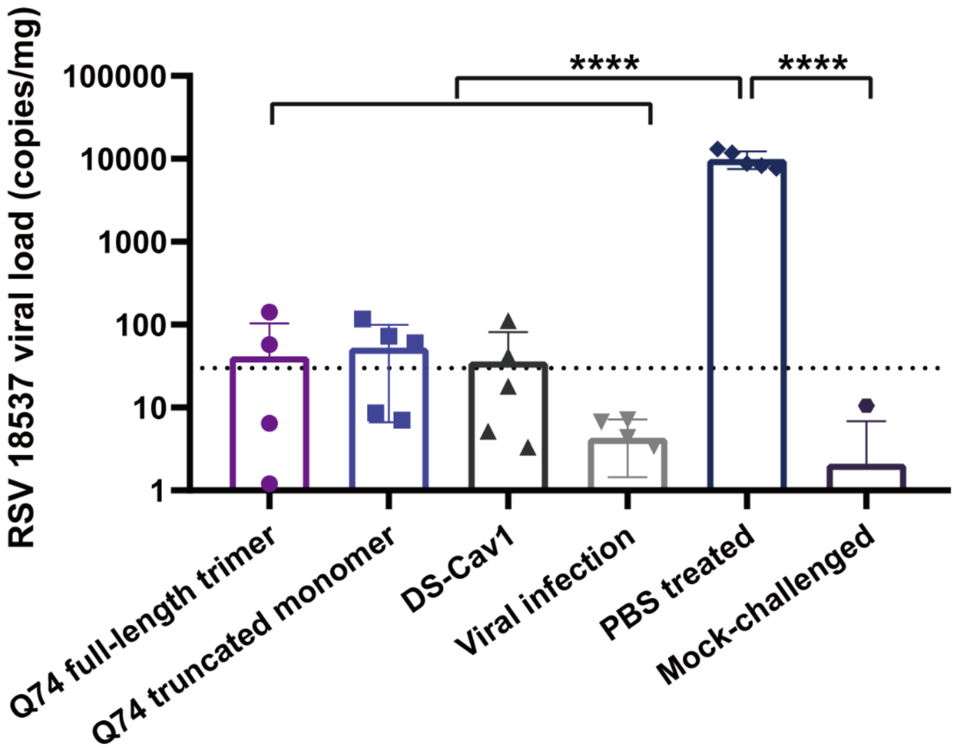

Figure S6. Protective efficacy of RSV F immunogens against RSV 18537 virus challenge., related to Figure 5. RSV viral load (RSV N gene copies) in the lung tissue after RSV 18537 virus challenge. Three weeks after immunization, mice were challenged with 5×10^5 PFU RSV 18537 virus at weeks 7. PBS treated animals served as viral infection model controls, mock-challenged animals served as non-viral infection model controls. Viral load was measured four days after challenge in the lung tissue via RT-qPCR and are reported as copies/mg within each group as mean ± SD. Statistical comparisons were performed using one-way analysis of variance (ANOVA) between different groups. * means P < 0.05; ** means P < 0.01 ; *** means P < 0.001; **** means P < 0.0001; LOD = limit of detection at 30 copies/mg.

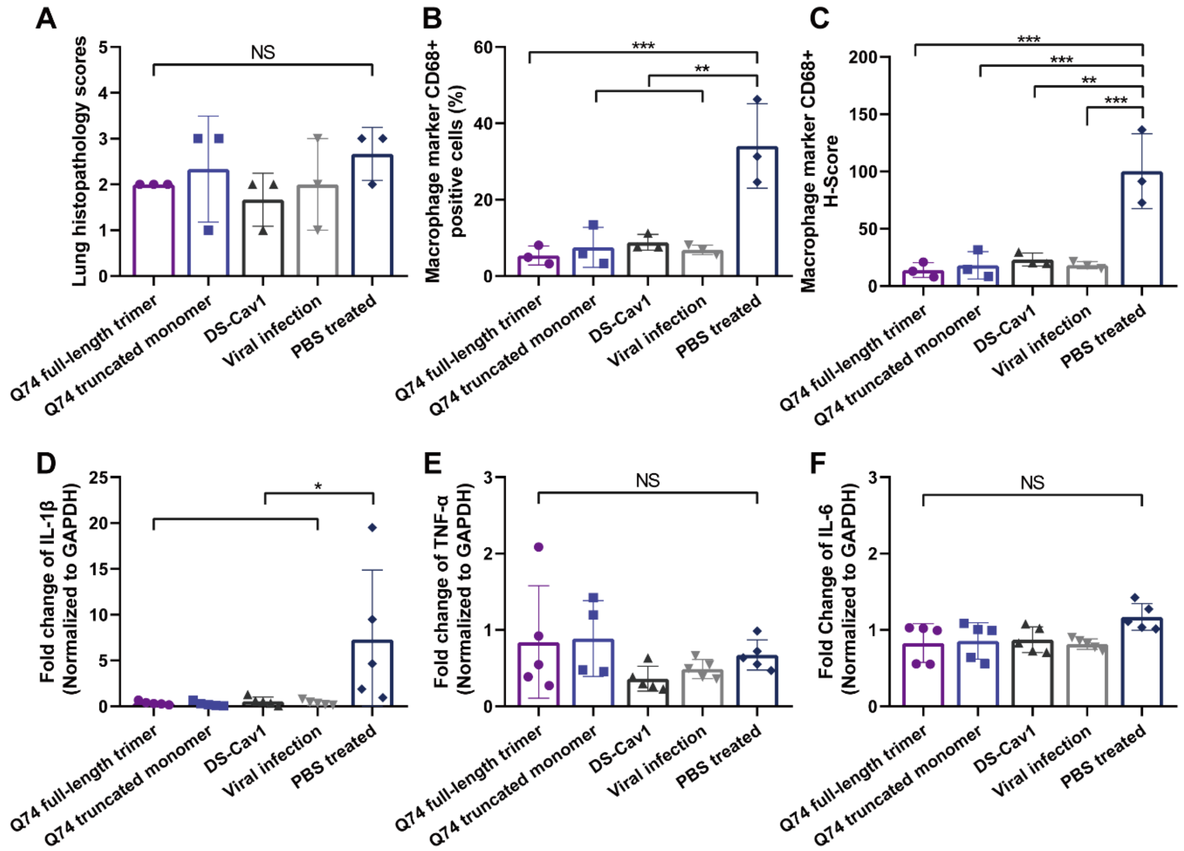

Figure S7. RSV infection induced lung inflammation, related to Figure 5. (A) Histopathological scores of RSV-induced lung tissue damage/inflammation. (B, C) Immunohistochemical analysis of $CD68^+$ macrophages: (B) Percentage of $CD68^+$ cells; (C) CD68 H-Scores. (D-F) Lung mRNA expression levels of proinflammatory cytokines: (D) IL-1β, (E) TNF-α, (F) IL-6, measured by RT-qPCR.

Table S1. Amino acid sequences of RSV F immunogens, related to Figure 1.

Design number	Sequence
Q74 full-length trimer	MELLILKANAITTILTAVTFCFASGQNITEEFYQSTCSAVSKGYLSAL<br>RTGWYTSVITIELSNIKENKCNGCDAKCKLIKQELDKYLNAVTELQ<br>LLMQSTPATNNGGSGGSGGSAIASGVAVSKVLHLEGEVNKIKSALLST<br>NKAVVSLSNGVSVLTSKVLDLKNYIDKQLLPIVNKQSCSISNIETVIEF<br>QQKNNRLLEITREFSVNAGVTTPVSTYMLTNSELLSLINDMPITNDQ<br>KKLMSNNVQIVRQQSYSIMSIIKEEVLAYVVQLPLYGVIDTPCWKLH<br>TSPLCTTNTKEGSNICLTRTDRGWYCDNAGSVSFFPQAETCKVQSNR<br>VFCDTMNSLTLPSEVNLCNVDIFNPKYDCKIMTSCTDVSSSVITSLGA<br>IVSCYGKTKCTASNKNRGIIKTFSNGCDYVSNKGVDTVSVGNTLYYV<br>NKQEGKSLYVKGEPIINFYDPLVFPCDEFDASISQVNEKINQSLAFIR<br>KSDELLSAIGGYIPEAPRDGQAYVRKDGEWVLLSTFL*
Q74 truncated monomer	MELLILKANAITTILTAVTFCFASGQNITEEFYQSTCSAVSKGYLSAL<br>RTGWYTSVITIELSNIKENKCNGCDAKCKLIKQELDKYLNAVTELQ<br>LLMQSTPATNNGGSGGSGGSAIASGVAVSKVLHLEGEVNKIKSALLST<br>NKAVVSLSNGVSVLTSKVLDLKNYIDKQLLPIVNKQSCSISNIETVIEF<br>QQKNNRLLEITREFSVNAGVTTPVSTYMLTNSELLSLINDMPITNDQ<br>KKLMSNNVQIVRQQSYSIMSIIKEEVLAYVVQLPLYGVIDTPCWKLH<br>TSPLCTTNTKEGSNICLTR*
DS-Cav1	MELLILKANAITTILTAVTFCFASGQNITEEFYQSTCSAVSKGYLSAL<br>RTGWYTSVITIELSNIKENKCNGTDAKVKLIKQELDKYKNAVTELQ<br>LLMQSTPATNNRARRELPRFMNYTLNNAKKTNVTLSKKRKRRFLG<br>FLLGVGSAIASGVAVCKVLHLEGEVNKIKSALLSTNKAVVSLSNGVSV<br>LTFKVLDLKNYIDKQLLPILNKQSCSISNIETVIEFQQKNNRLLEITR<br>EFSVNAGVTTPVSTYMLTNSELLSLINDMPITNDQKKLMSNNVQIVR<br>QQSYSIMCIIKEEVLAYVVQLPLYGVIDTPCWKLHTSPLCTTNTKEG<br>SNICLTRTDRGWYCDNAGSVSFFPQAETCKVQSNRVFCDTMNSLTL<br>PSEVNLCNVDIFNPKYDCKIMTSKTDVSSSVITSLGAIVSCYGKTKCT<br>ASNKNRGIIKTFSNGCDYVSNKGVDTVSVGNTLYYVNKQEGKSLYVK<br>GEPIINFYDPLVFPSDEFDASISQVNEKINQSLAFIRKSDELLSAIGGYIP<br>EAPRDGQAYVRKDGEWVLLSTFL*

Underline, signal sequence; brown, F2 region; green, F1 region; purple, T4 fibritin-trimerization foldon; red with underlined, variant
