# Supplementary material for: Immunofocusing design of a fusion glycoprotein monomer vaccine for respiratory syncytial virus
Source: Mol Ther. 2025 Jul 28;33(11):5401–14. doi: 10.1016/j.ymthe.2025.07.040 (PMC12628061; doi:10.1016/j.ymthe.2025.07.040)
Supplement: Document S2. Article plus supplemental information [file mmc2.pdf]

Molecular Therapy
Original Article

Immunofocusing design of a fusion glycoprotein monomer vaccine for respiratory syncytial virus

Qianqian Li,[1,2,3,4] Zhiming Li,[1,4] Jiamin Chen,[1,4] Qiuju He,[1] Hongjian Xiao,[1] Haiwei Li,[1] Huan Li,[1] Heng Zhang,[1] Yaoyun Yang,[1] Rong Bi,[1] Zichen Li,[1] Song Xiao,[1] Yanwei Bi,[1] Bingyan Liang,[1] Luxia Huang,[1] Mengyi Zhang,[1] Jincheng Tong,[1] Haoyue Long,[1] Ru Li,[1] Jinmei Duan,[1] Zhihua Li,[1,2] and Youchun Wang[1,2,3]

[1]Institute of Medical Biology, Chinese Academy of Medical Sciences and Peking Union Medical College, Kunming 650118, China; [2]State Key Laboratory of Respiratory Health and Multimorbidity, Institute of Medical Biology, Chinese Academy of Medical Sciences and Peking Union Medical College, Kunming 650118, China; [3]Key Laboratory of Pathogen Infection Prevention and Control, Ministry of Education, Institute of Medical Biology, Chinese Academy of Medical Sciences and Peking Union Medical College, Kunming 650118, China

Respiratory syncytial virus (RSV) can cause severe lower respiratory tract infections in infants and the elderly. Previously, most RSV vaccine antigen design strategies have focused on the stability of the prefusion (PreF) protein trimer conformation. We hypothesized that the RSV monomer might serve as a vaccine candidate immunogen. Here, we constructed an immunofocusing-based RSV F protein Q74 truncated monomer by truncating the RSV F protein and retaining only the head and neck regions. The structural prediction and protein antigenicity characterization of Q74 truncated monomer both indicated that it could bind to the antigenic site Ø-specific antibody D25, suggesting that this immunogen retained the structural features of PreF. In addition, the Q74 truncated monomer not only induced neutralizing and binding antibodies with a strength comparable to that of DS-Cav1 but also focused the immune response on antigenic site Ø and site II. In summary, we found that the RSV F protein monomer designed based on immunofocusing could induce relative immunogenicity and immune protection, providing a design strategy for RSV vaccines.

INTRODUCTION

Respiratory syncytial virus (RSV) is an enveloped virus belonging to the Pneumovirus genus of the Paramixoviridae family.[1] RSV infection can lead to increased mucus secretion, airway inflammation, and narrowed airways.[2] It is an important pathogen that causes lower respiratory tract infections such as bronchitis, bronchiolitis, and pneumonia in children,[3] older adults, and immunocompromised persons.[4] Consequently, there is an urgent necessity to develop RSV vaccines to safeguard the health of both the elderly and children.[5] The RSV F protein is highly conserved in RSV A and B strains and is a major target protein for vaccine development.[6] The RSV F protein is a typical type I transmembrane protein, which contains 574 amino acids and exists in a trimeric form. The RSV F protein has 2 structural conformations and changes from the prefusion state (PreF)[7] to the postfusion state (PostF)[8] during membrane fusion. The PostF conformation is stable, while the PreF is metastable until stabilizing mutations are introduced. The RSV F protein has at least 6 antigenic sites. Antibodies against sites Ø and V, which are only present on PreF, have strong neutralizing activity, indicating that PreF can induce such antibodies.[9] Thus, a stable PreF conformation is crucial for vaccine research.[10]

Structure-based antigenic rational design is a key to RSV subunit vaccine development.[11] Multiple amino acid-modification strategies, such as the addition of engineered disulfide bonds,[12] dityrosine bonds,[13] cavity hydrophobic filling mutations,[12] proline mutations,[14,15] electrostatic interaction mutations,[16] and introduction of trimerization domains,[17] can stabilize the RSV F protein in the PreF conformation and have been applied in RSV vaccine research. In a pioneering work, an S155C-S290C disulfide bond and 2 cavity-filling mutations, S190F and V207L, were used to generate a PreF stabilized protein named DS-Cav1, which is often used as a comparison standard for subsequent antigen development.[12,18] The Arexvy recombinant protein vaccine, Abrysvo recombinant protein vaccine,[19] and mRNA vaccine all stabilize the RSV PreF conformation through amino acid modifications and have all been successfully marketed,[20] thus demonstrating the efficacy and safety of the PreF trimer immunogen.

Currently, developments in RSV recombinant protein vaccine focus on the design of protein trimer immunogens and the stabilization of the PreF protein conformation. However, the use of exogenous trimer motifs may generate immune responses against this protein

Received 21 January 2025; accepted 24 July 2025;
https://doi.org/10.1016/j.ymthe.2025.07.040.

[4]These authors contributed equally

Correspondence: Youchun Wang, Institute of Medical Biology, Chinese Academy of Medical Sciences and Peking Union Medical College, Kunming 650118, China.
E-mail: wangyc@imbcams.com.cn

Published by Elsevier Inc. on behalf of The American Society of Gene and Cell Therapy.
This is an open access article under the CC BY license (http://creativecommons.org/licenses/by/4.0/).

fragment that are irrelevant to RSV protection.[21] Here, we proposed a hypothesis whether the RSV F protein monomer can induce good immunogenicity and immunoprotection. In addition, immunofocus-based designs of SARS-CoV-2 receptor-binding domain (RBD) immunogens,[22] Middle East respiratory syndrome-CoV RBD fragments,[23] Ebola virus GP immunogens,[24] and influenza hemagglutinin RBD[25] all demonstrated excellent protective effects. This indicates that deleting or masking irrelevant epitopes of immunogens enhances and focuses effective immune responses.[26] There are differences in the immune responses induced by different epitopes of the RSV F protein. Specifically, site Ø can induce strong neutralizing antibodies, while the neutralizing antibodies induced by site IV have weak activity.[27] Studies have shown that good immunogenicity can be achieved through the truncation and modification of the F protein.[28] Therefore, we further hypothesize that an RSV F protein monomer based on immunofocusing design can induce a more effective immune response.

Here, we have designed a truncated RSV F protein that retains only the head and neck regions and introduces mutations that were previously found to stabilize the PreF conformation. This immunogen can achieve immunofocusing and the monomer is easy to produce, purify, and store. The RSV F monomer based on immunofocusing design provides another effective strategy, which can expand the development methods of RSV F protein vaccines.

RESULTS

Immunofocusing design strategies for RSV F Q74 immunogen

RSV PreF structure can be divided into 3 regions: the RSV "head" region, which comprises antigenic site Ø, is highly neutralization sensitive and exclusive to the PreF conformation; the RSV "neck" region, which comprises antigenic site II and site V; and the RSV "stem" region, which comprises antigenic site IV and site I; these regions are recognized by antibodies with lower potency (Figure 1A). We hypothesized that antigenic design against the head and neck regions of the F protein can focus the immune response, thereby inducing higher immunogenicity. Therefore, we designed the Q74 truncated monomer immunogen and Q74 full-length trimer immunogen to verify whether immunofocusing design strategies and protein monomers could induce protection against RSV (Table S1).

Q74 truncated monomer immunogen is an immunofocusing design that has no trimerization domain and comprised F2 residues 26–105 linked to F1 residues 147–336 by a GGSGGSGGS linker (Figure 1B). In contrast to the Q74 full-length trimer, the Q74 truncated monomer lacks residues 337–513 in the F1 subunit and does not include the C-terminal T4-foldon trimerization domain. Q74 truncated monomer immunogen contains T72C-V76C disulfide bond mutation and K87L cavity-filling mutation, which were found to stabilize the RSV PreF conformation in previous studies. The Q74 full-length trimer immunogen contains the RSV head region, neck region, and stem region, which comprised F2 residues 26–105 linked to F1 residues 147–513 by a GGSGGSGGS linker. In addition, a bacteriophage T4-foldon trimerization domain is connected at the C terminus of F1 by an SAIG linker (Figure 1C). This T4-foldon domain serves as a critical element that enables the protein to adopt a trimeric conformation. The Q74 full-length trimer immunogen contains T72C-V76C disulfide bond mutation, K399C-S485C disulfide bond mutation, and K87L cavity-filling mutation, which were found to stabilize the RSV PreF conformation in previous studies. DS-Cav1 is a PreF-stabilized protein, which contains a S155C-S290C disulfide bond and 2 cavity-filling mutations (S190F and V207L). DS-Cav1 was made up of F2 residues and F1 residues (amino acids [aa] 26–513), which are connected at the C terminus of F1 to a T4-foldon domain (Figure 1D).

Using AlphaFold3 prediction, we modeled the interaction structures of immunogens Q74 truncated monomer (Figure 1B), Q74 full-length trimer (Figure 1C), and DS-Cav1 (Figure 1D), each with monoclonal antibodies (mAbs) D25 and 101F. The predicted results showed that the structure of the Q74 truncated monomer is similar to that of the RSV PreF head and neck regions. In addition, the Q74 truncated monomer could only bind to mAb D25 and not to mAb 101F. However, the structure of the Q74 full-length trimer is similar to that of the RSV PreF. In addition, the Q74 full-length trimer and DS-Cav1 can bind to mAb D25 through antigenic site Ø and to mAb 101F through antigenic site IV.

Antigenic and physical properties of RSV F Q74 immunogen

Q74 truncated monomer, Q74 full-length trimer, and DS-Cav1 were expressed in Expi293F cells. After being transfected with the plasmid, Expi293F cells were cultured for 5 days. The culture supernatant was then harvested and followed by a 2-step affinity chromatography process to obtain the purified immunogens. Q74 truncated monomer had a calculated molecular weight of 33 kDa and migrated as a comparable molecular weight band under reduced SDS-PAGE conditions and western blot analysis using anti-RSV mAb D25 and motavizumab (Figure S1, lane 2). Moreover, the actual bands under reduced SDS-PAGE conditions (monomer) and western blot analysis of Q74 full-length trimer (Figure S1, lane 1) and DS-Cav1 (Figure S1, lane 3) matched the calculated molecular weight of Q74 full-length trimer (56 kDa × 3) and DS-Cav1 (60 kDa × 3). In addition, the primary structures of the purified Q74 truncated monomer and Q74 full-length trimer were confirmed by peptide mapping analysis, which showed that only the N-terminal signal peptide region and the remaining 17 aa were not detected (Figure S2).

To confirm the antigenic properties of RSV immunogens, we first tested the binding abilities of purified immunogens to specific mAbs by indirect enzyme-linked immunosorbent assay (ELISA). Antibodies targeting site Ø (D25 and AM22) bind exclusively to the PreF protein. Antibodies targeting site II (palivizumab and motavizumab) and site IV (101F) exhibit binding to both PreF and PostF conformations. In contrast, the site I-specific antibody 4D7 demonstrates markedly stronger binding affinity for PostF compared to PreF. The experimental results showed that the 3

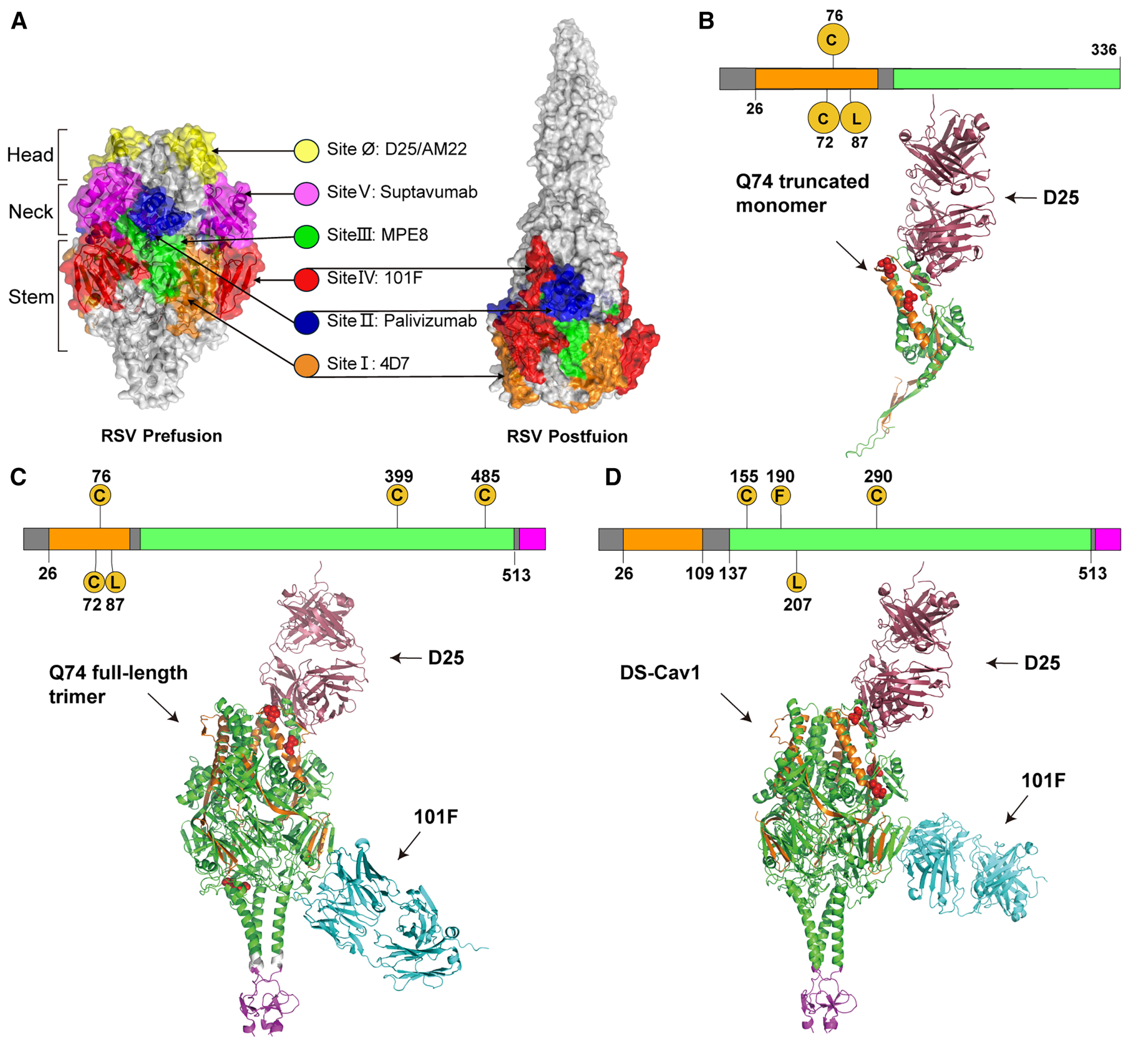

Figure 1. Design and structure modeling of RSV F immunogens

(A) RSV F antigenic sites. RSV PreF protein (PDB: 7LUC) and PostF protein (PDB: 3RRT) conformations are visualized using Pymol. Antigenic sites are colored for each structure. Site I is colored orange, site II is colored dark blue, site III is colored green, site IV is colored red, site V is colored purple, and site Ø is colored yellow. Respective mAbs for each site are shown. (B–D) Models of RSV F immunogens Q74 truncated monomer (B), Q74 full-length trimer (C), and DS-Cav1 (D) with D25 antibody (colored dark purple) and 101F antibody (colored blue), predicted by the AlphaFold3 multimer approach. For each immunogen an illustration of the genetic construct (top) and a ribbon diagram (bottom) are depicted. F_2 is colored orange, F_1 is colored green, and the T4-foldon domain is colored purple. The numbers denote the amino acid positions within the sequence.

immunogens, Q74 truncated monomer, Q74 full-length trimer, and DS-Cav1, had essentially the concentration-dependent binding to mAb D25 (site Ø, Figure 2A), AM22 (site Ø, Figure 2B), palivizumab (site II, Figure 2C), and motavizumab (site II, Figure 2D). However, the binding of Q74 truncated monomer to mAb D25, AM22, and palivizumab was weaker compared to the trimerized proteins, possibly due to its monomeric form. In addition, the Q74 full-length trimer and DS-Cav1 were capable of binding to mAb 101F (site IV), whereas the Q74 truncated monomer did not react due to the absence of the stem region (Figure 2E). It was observed that only the DS-Cav1 immunogen exhibits binding to mAb 4D7 (site I), which implies that both the Q74 truncated monomer and the Q74 full-length trimer assume a more stable PreF conformation (Figure 2F). Consistent results were obtained using the sandwich ELISA. The results of RSV-specific mAb motavizumab/D25-horseradish peroxidase (HRP) sandwich ELISA showed that the 3 immunogens had similar antibody binding curves and all of them had antigenic site II and site Ø (Figure 2G). Moreover, the binding curve of the Q74 truncated monomer decreased more slowly compared to the trimerized protein, indicating that the Q74 truncated monomer was able to better demonstrate the antigens site II and site Ø. However, the results of RSV-specific mAb motavizumab/101F-HRP sandwich ELISA showed that Q74 full-length trimer and DS-Cav1 had similar antibody

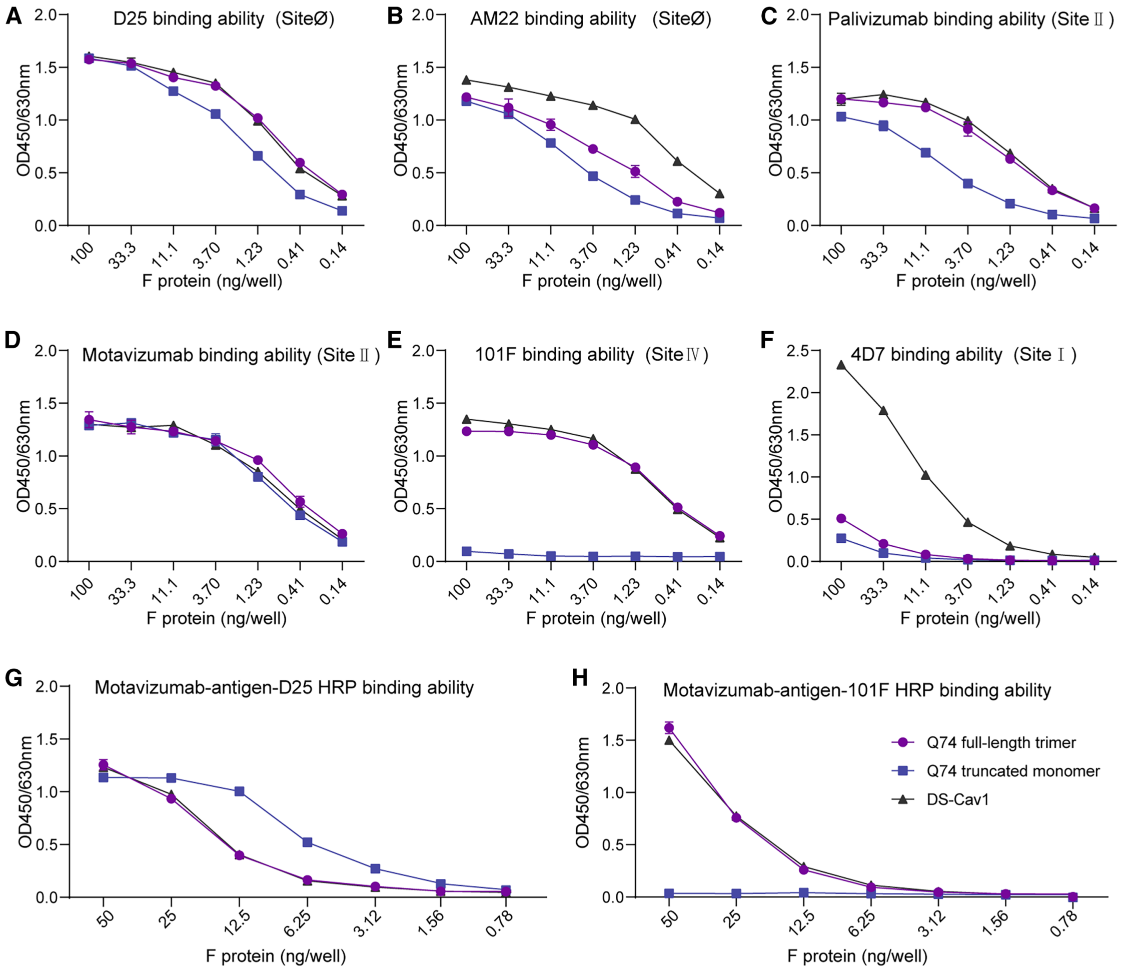

Figure 2. Antigenic properties of RSV F immunogens

(A–F) The concentration-dependent binding abilities of site Ø antibody D25 (A), site Ø antibody AM22 (B), site II antibody palivizumab (C), site II antibody motavizumab (D), site IV antibody 101F (E), and site I antibody 4D7 (F) to Q74 full-length trimer and Q74 truncated monomer were investigated by indirect ELISA. (G and H) Detection of Q74 full-length trimer and Q74 truncated monomer using RSV-specific mAb motavizumab/D25-HRP pair (G) and mAb motavizumab/101F-HRP pair (H) by sandwich ELISA. The binding abilities of DS-Cav1 were detected for comparison. Data are presented as mean ± SD for duplicate wells. The experiments are repeated 3 times, and the results of 1 independent experiment are shown here.

binding curves and both had antigenic site II and site IV, while Q74 truncated monomer did not have antigenic site IV (Figure 2H).

To confirm the physical properties of RSV immunogens, we first evaluated the physical stability of purified immunogens by quantifying the effect of high-temperature stress, extreme pH stress, and freeze-thaw cycles on recognition via motavizumab/D25-HRP, motavizumab/AM22-HRP, and motavizumab/101F-HRP sandwich ELISA. The results indicated that all 3 proteins retained conformational integrity following 1-h incubation at 50°C and 60°C. However, complete loss of PreF conformation was observed for all constructs upon exposure to 70°C. Notably, the Q74 truncated monomer maintained structural stability through 3 and 5 freeze-thaw cycles, whereas both the Q74 full-length trimer and DS-Cav1 exhibited significantly diminished epitope-binding capacity after repeated freeze-thaw treatments. Acidic (pH 3.5) and alkaline (pH 10) buffer exposure for 1 h demonstrated minimal impact on protein conformation across all tested samples (Figure 3A). We further used nano-differential scanning fluorimetry (nanoDSF) recording changes in tryptophan fluorescence (ratio of 350:330 nm) during protein denaturation. Then, the first derivative of the ratio of 350:330 nm was used to determine their melting temperature (Tm) of different transition states (Figure 3B). The results indicated that the onset conformational change temperature of Q74 full-length trimer was 42.9°C, with 2 structural alterations during heating. The Tm values were 62.7°C for the first and 83.1°C for the second. The Q74 truncated monomer had an onset temperature of 62.0°C and a Tm of 66.3°C, whereas DS-Cav1 exhibited an onset temperature of 27.1°C, a Tm1 of 63.5°C, and a Tm2 of 77.9°C. Therefore, compared to DS-Cav1, the higher onset temperature and Tm value

A

		Heat stress			Freeze-thaw			pH	
	Immunogen	50°C	60°C	70°C	1	3	5	3.5	10
Motavizumab-Ag-D25 HRP	Q74 full-length trimer	0.9	0.8	0.0	0.6	0.2	0.1	0.9	1.3
	Q74 truncated monomer	0.9	0.8	0.0	1.0	1.3	1.2	0.7	1.1
	DS-Cav1	0.8	0.8	0.0	0.3	0.1	0.0	0.7	1.1
Motavizumab-Ag-AM22 HRP	Q74 full-length trimer	1.0	0.7	0.0	0.7	0.2	0.1	1.0	1.1
	Q74 truncated monomer	0.9	0.8	0.0	1.1	1.0	1.0	0.6	1.1
	DS-Cav1	0.8	0.7	0.0	0.4	0.1	0.0	0.6	1.1
Motavizumab-Ag-101F HRP	Q74 full-length trimer	1.0	0.5	0.1	0.6	0.2	0.1	0.7	1.0
	Q74 truncated monomer	NA	NA	NA	NA	NA	NA	NA	NA
	DS-Cav1	0.9	0.9	0.1	0.4	0.2	0.0	0.9	1.2

B

	Ratio 350nm/330nm					
Thermal stability	Tm1/°C		Tm2/°C		ON/°C	
Immunogen		σ		σ		σ
Q74 full-length trimer	62.7	0.12	83.1	0.14	42.9	0.52
Q74 truncated monomer	66.3	0.08	NA	NA	62.0	0.34
DS-Cav1	63.5	0.11	77.9	0.09	27.1	0.71

Figure 3. Physical properties of RSV F immunogens

(A) Physical stability of immunogens refers to motavizumab-Ag-D25, motavizumab-Ag-AM22, and motavizumab-Ag-101F binding abilities retained after exposure to heat stress (1 h at 50°C, 60°C, and 70°C), freeze-thaw (1, 3, and 5 cycles), and pH stress (1 h at pH 3.5 and pH 10.0). Higher ratios mean that the protein is more stable, while lower ratios mean that the protein undergoes a conformational change. N/A, not applicable. (B) Melting temperature (Tm) determination for Q74 full-length trimer, Q74 truncated monomer, and DS-Cav1 (1 mg/mL) were determined by nanoDSF. Onset temperature of Q74 full-length trimer, Q74 truncated monomer, and DS-Cav1 were 42.9°C, 62.0°C, and 27.1°C, respectively. Tm1 values of Q74 full-length trimer, Q74 truncated monomer, and DS-Cav1 were 62.7°C, 66.3°C, and 63.5°C, respectively. These values are from 3 different measurements.

of the Q74 truncated monomer suggest greater stability under normal storage conditions.

Immunization with Q74 truncated monomer induces immune responses in mice

To confirm immunogenicity, we evaluated the serum-binding and neutralizing antibodies against RSV A and B strains in mice immunized with Q74 truncated monomer, Q74 full-length trimer, and DS-Cav1. At week 0, all mice were initially infected with 5×10^5 plaque-forming units (PFU) RSV A2 virus via intranasal administration to mimic the background of RSV infection in the elderly. Subsequently, at week 4, the mice were either immunized with 10 μg Q74 truncated monomer, Q74 full-length trimer, or DS-Cav1 immunogen, and each was adjuvanted with 50 μg aluminum hydroxide through intramuscular injection or underwent a second nasal infection with RSV A2 virus (Figure 4A).

Serum-binding antibody titers to RSV PreF were assessed at weeks 4, 7, and 10 (Figure 4B). Weak binding antibodies against the PreF immunogen were detected in the serum at the fourth week after RSV infection. At week 7, sera from mice immunized with the Q74 full-length trimer, Q74 truncated monomer, and DS-Cav1 exhibited significantly higher levels of PreF-binding antibodies compared to those with viral infection (geometric mean titer [GMT] = 234,140, 51,200, 117,070, and 11,143, respectively). At week 10, sera from mice immunized with the Q74 full-length trimer, Q74 truncated monomer, and DS-Cav1 continued to exhibit significantly higher levels of PreF-binding antibodies compared to those with viral infection (GMT = 135,118, 38,802, 117,627, and 12,800, respectively). Serum-binding antibody titers to RSV PostF were assessed at weeks 4, 7, and 10 (Figure 4C). Weak binding antibodies against the PostF immunogen were detected in the serum at the fourth week after RSV infection. At week 7, sera from mice immunized with the Q74 full-length trimer and DS-Cav1 exhibited significantly higher levels of PostF-binding antibodies compared to those with Q74 truncated monomer and viral infection (GMT = 1,401,355, 562,463, 117,627, and 58,813, respectively). At week 10, sera from mice immunized with the Q74 full-length trimer and DS-Cav1 continued to exhibit significantly higher levels of PostF-binding antibodies compared to those with Q74 truncated monomer and viral infection (GMT = 713,155, 470,507, 51,200, and 44,572, respectively). The ratio of serum-binding responses to RSV PreF and PostF were assessed at weeks 4, 7, and 10 (Figure 4D).

Neutralizing antibody titers using the live-virus A2 strain neutralization assay were evaluated (Figure 4E). Weak neutralizing antibodies against the RSV A2 strain were detected in the serum at the fourth week. However, at week 7, no significant difference was observed in the neutralizing antibody titers of the immune sera against the RSV A2 strain, with median effective concentration (EC_{50}) values of 1,270, 439, 1,008, and 579, respectively. At week 10, no significant differences were observed in the neutralizing antibody titers of the immune sera against the RSV A2 strain, with EC_{50} values of 877, 348, 635, and 419, respectively. Neutralizing antibody titers using live-virus 18537 strain neutralization assay were evaluated (Figure 4F). Weak neutralizing antibodies against the RSV 18537 strain were detected in the serum at week 4. In addition, at week 7, neutralizing antibody titers against RSV B18537 strain varied, with EC_{50} values of 389, 126, 579, and 40, respectively. At week 10, no significant differences were observed in the neutralizing antibody titers of the immune sera against RSV B18537 strain, with EC_{50} values of 95, 57, 230, and 25, respectively. The ratio of serum-neutralizing responses to RSV A2 and B18537 strains were assessed at weeks 4, 7, and 10 (Figure 4G).

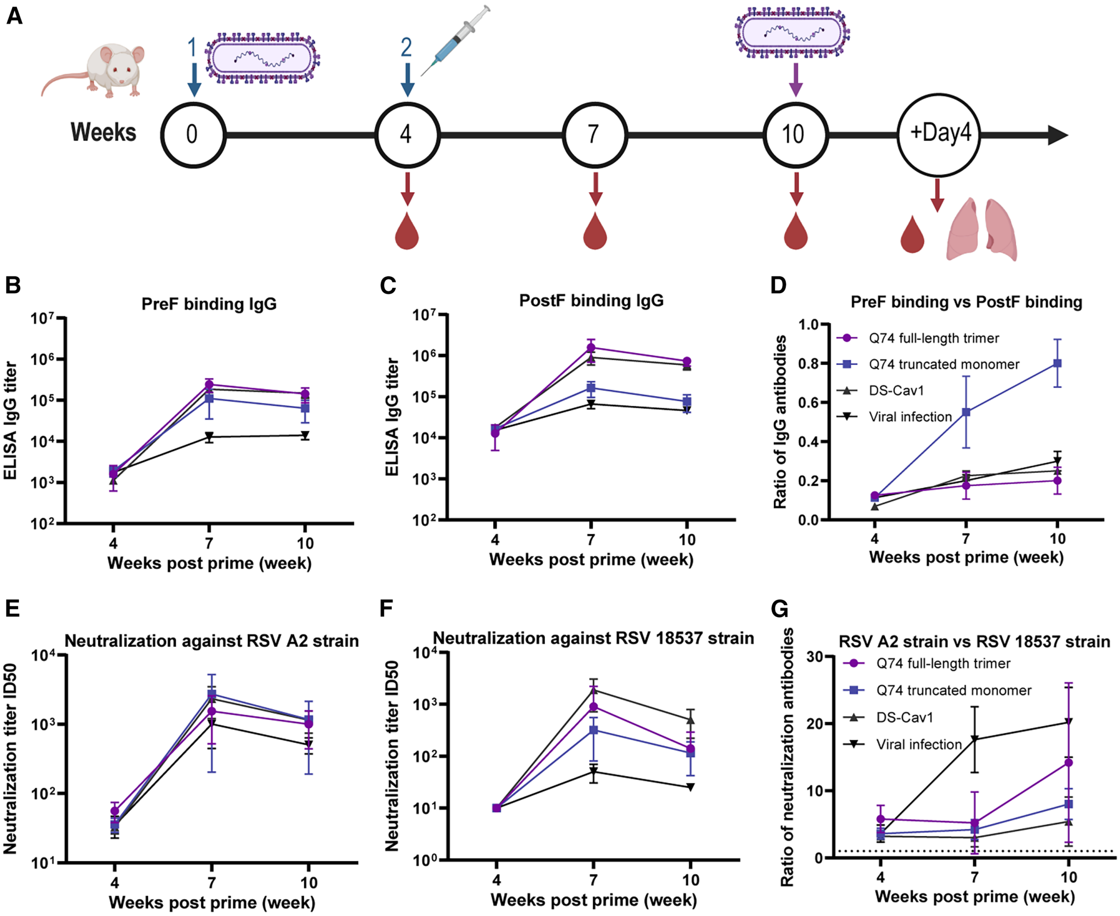

Figure 4. Immunogenicity evaluation of RSV F immunogens in mice

(A) Procedures of immunization, viral challenge, and sample collection. Created with BioRender software. All mice (5/group) were first infected with 5×10^5 PFU RSV A2 virus by nasal application at week 0 and then immunized with each immunogen at week 4 by intramuscular injection. RSV viral infection animals were infected with RSV A2 virus twice at weeks 0 and 4. Sera were collected at weeks 4, 7, and 10. (B–D) Binding antibody titers using ELISA assay were evaluated. (B) Serum-binding responses to RSV PreF were assessed at weeks 4, 7, and 10. (C) Serum-binding responses to RSV PostF were assessed at weeks 4, 7, and 10. (D) The ratio of serum-binding responses to RSV PreF and PostF were assessed at weeks 4, 7, and 10. (E–G) Neutralizing antibody titers using live-virus neutralization assay were evaluated. (E) Serum-neutralizing responses to RSV A2 strain were assessed at weeks 4, 7, and 10. (F) Serum-neutralizing responses to RSV 18537 strain were assessed at weeks 4, 7, and 10. (G) The ratio of serum-neutralizing responses to RSV A2 and B18537 strains were assessed at weeks 4, 7, and 10. Titers are reported as the 50% geometric mean titer (GMT) within each group as mean ± SD. GMTs were log10 transformed, and statistical comparisons were performed using 1-way analysis of variance (ANOVA) between different groups. *$p < 0.05$; **$p < 0.01$; ***$p < 0.001$; ****$p < 0.0001$; ns, not significant.

To corroborate the observations, an independent immunization study with enhanced statistical power (n = 15 mice/group) was conducted. Analysis of the complete dataset (Figure S3) revealed that for RSV A2 neutralization (Figure S3A), Q74 monomer elicited comparable titers to DS-Cav1 ($p > 0.05$). Against the RSV 18537 strain (Figure S3B), Q74 monomer and trimer vaccines generated significantly higher neutralizing titers than the live-virus control group, although no statistically significant differences were observed between Q74 monomer and DS-Cav1 immunogens.

Bronchoalveolar lavage fluid (BALF) samples from different immunization groups were subjected to 5-fold dilution, and immunoglobulin A (IgA) levels were quantified using ELISA. The results demonstrate that groups receiving 2 rounds of viral infection exhibited significantly stronger mucosal immune responses ($p < 0.0001$). In contrast, other vaccine-immunized groups induced only modest mucosal immunity, with IgA optical density (OD) values approximately 3-fold higher than negative mock controls (Figure S3C).

Additionally, cellular immunity data are provided in Figure S4. The levels of cytokine interferon-γ (IFN-γ) (Figure S4A), interleukin-2 (IL-2) (Figure S4B), and IL-4 (Figure S4C) secreted by splenic lymphocytes were measured by ELISA. The number of cytokine-secreting splenic lymphocytes were quantified by ELISpot (Figures S4D–S4F). These results confirm that the Q74 truncated monomer immunogen effectively induces robust cellular immunity.

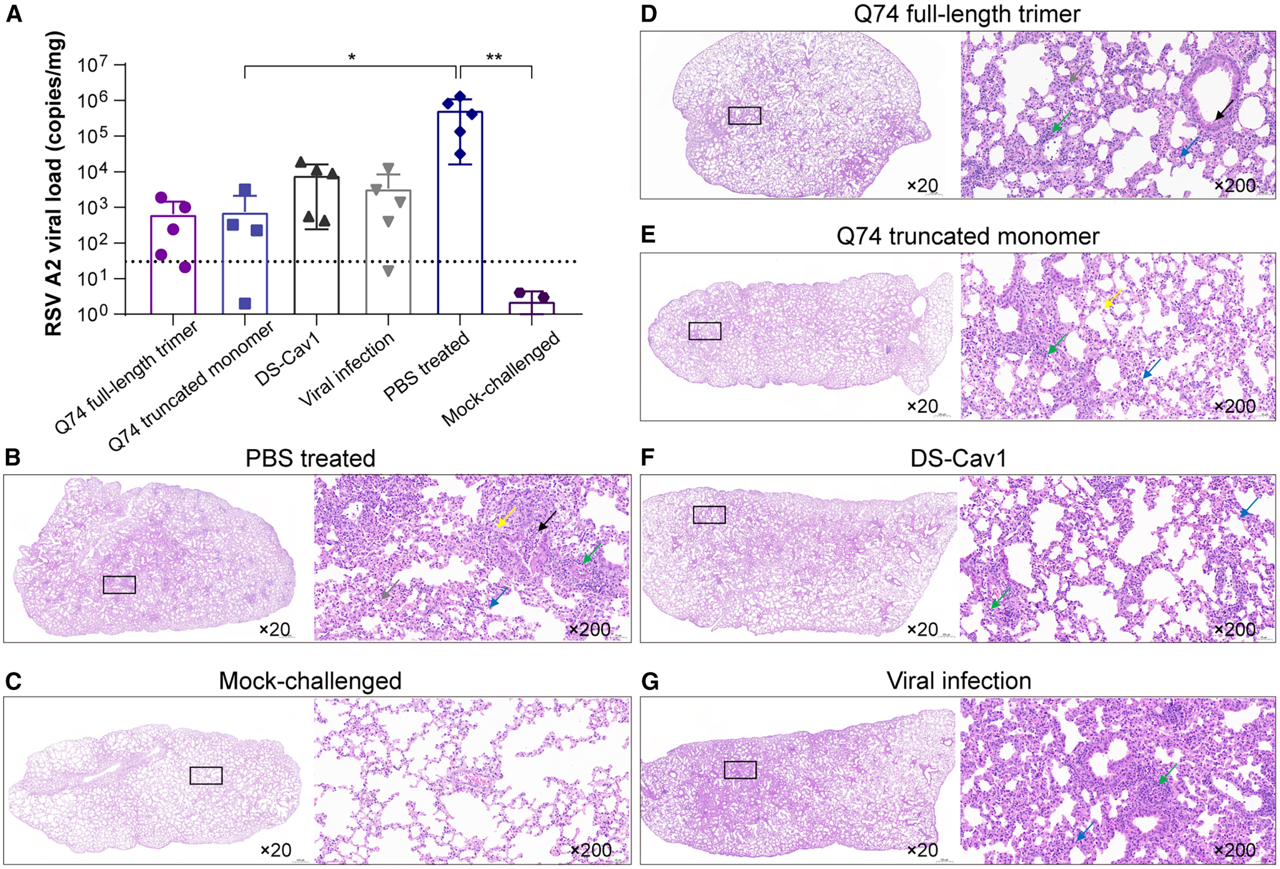

Figure 5. Protective efficacy of RSV F immunogens against RSV A2 virus challenge

(A) RSV viral load (RSV N gene copies) in the lung tissue after RSV A2 virus challenge. Mice were vaccinated as described above. Six weeks after immunization, mice were challenged with 5 × 10^5 PFU RSV A2 virus at week 10. PBS-treated animals served as viral infection model controls; mock-challenged animals served as non-viral infection model controls. Viral load was measured 4 days after challenge in the lung tissue via RT-qPCR and are reported as copies/mg within each group as mean ± SD. Statistical comparisons were performed using 1-way ANOVA between different groups. *$p < 0.05$; **$p < 0.01$; ***$p < 0.001$; ****$p < 0.0001$; Limit of detection at 30 copies/mg. (B–G) Representative hematoxylin and eosin-stained images of lung from each group at day 4 after virus challenge. General view of the lung (left, magnification 20×) along with histopathologic details from selected lung areas (black boxes) are displayed (right, magnification 200×). Green arrows indicate focal infiltration of lymphocytes, blue arrows indicate granulocytes, gray arrows indicate necrotic cell debris, yellow arrows indicate macrophages, and black arrows indicate necrotic cell debris and red blood cells in the lumen of bronchiole.

Immunization with Q74 truncated monomer provides protection against RSV-induced lung damage in mice

To evaluate protective efficacy, some mice were intranasally challenged with 5 × 10^5 PFU of the RSV 18537 strain virus at week 7 (Figure S6), and other mice were intranasally challenged with 5 × 10^5 PFU of RSV A2 strain virus at week 10 (Figure 5). PBS-treated and RSV A2 strain-challenged mice served as viral infection model controls; PBS mock-challenged mice were non-viral infection controls. Daily body weight is shown as a percentage of the weight on day 0 (Figure S5). On day 4 post-RSV infection, lung tissue viral loads were 641 copies/mg (Q74 full-length trimer), 743 copies/mg (Q74 truncated monomer), 8,078 copies/mg (DS-Cav1), 3,371 copies/mg (viral infection), and 538,897 copies/mg (PBS-treated and RSV A2 strain-challenged controls). Viral load upon challenge was reduced in mice vaccinated with the Q74 truncated monomer compared to PBS-treated mice (Figure 5A). The PBS-treated and RSV A2 strain-challenged mice exhibited typical moderate pathological changes in the lung, including focal inflammatory infiltration around the trachea and bronchi, epithelial cell shedding, and bleeding (Figure 5B). In contrast, the PBS mock-challenged mice showed no pathological change (Figure 5C). Histopathology revealed only slight pathological changes with local inflammatory infiltration in the lungs of mice immunized with the Q74 full-length trimer, Q74 truncated monomer, DS-Cav1, and those with viral infection (Figures 5D–5G). Histopathology of lung tissue damage/inflammation caused by RSV infection was quantified using a 4-tiered histopathological grading system (Figure S7A). The percentage of $CD68^+$ macrophages was quantified by immunohistochemistry (Figure S7B), and the histochemical scores (H-scores) were also quantified using the same method (Figure S7C). In addition, lung

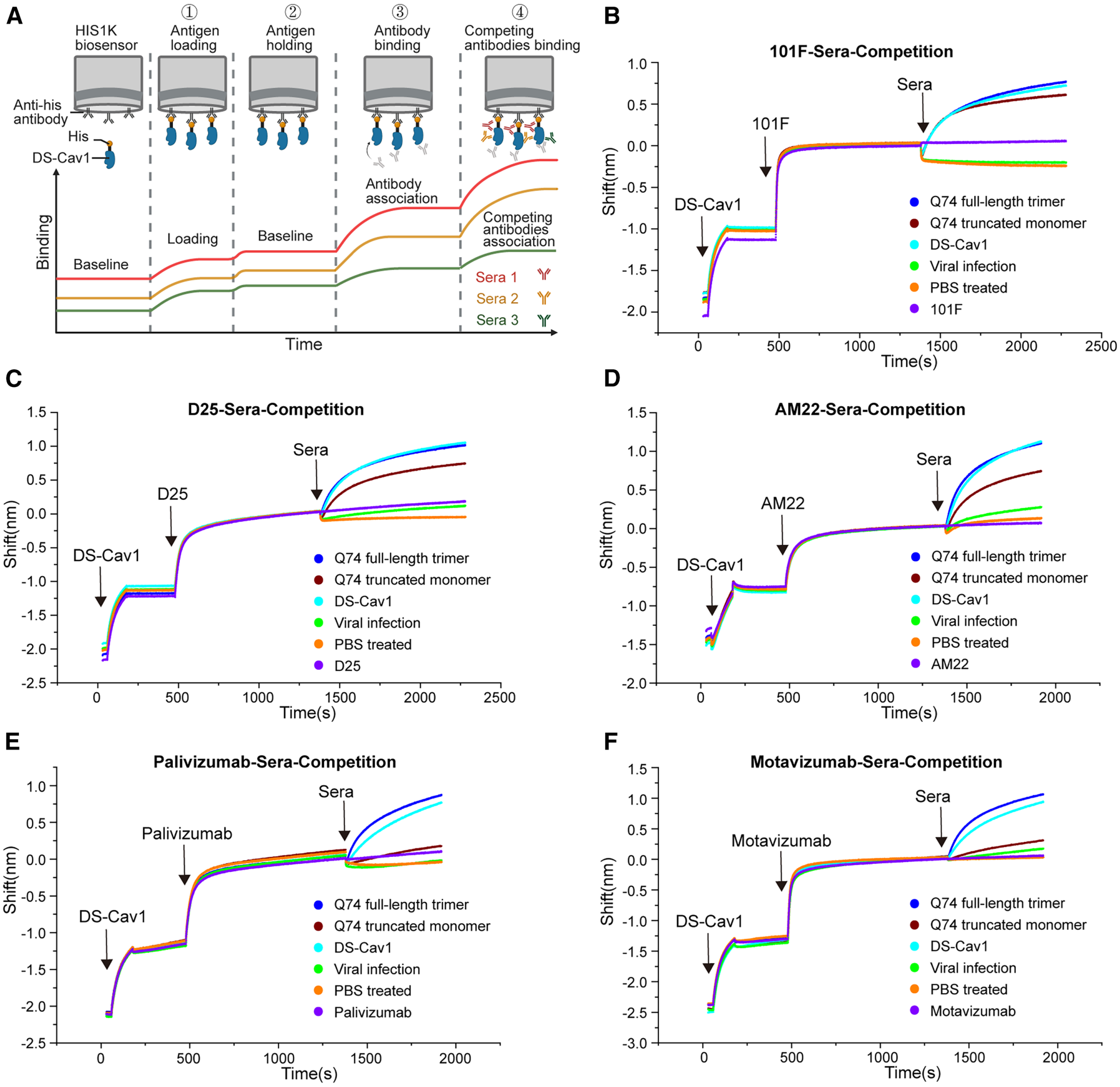

Figure 6. Antigenic site antibodies competition analysis of immunized sera

(A) A model depicting the experimental design of BLI-based competition experiments between antigenic site antibodies and RSV vaccine immunized sera. Created with BioRender software. (B–F) Data from the BLI-based competition experiments showing the binding of 5 different immunized sera (Q74 full-length trimer vaccine group, Q74 truncated monomer vaccine group, DS-Cav1 vaccine group, Vial infection group, and PBS-treated group) after blocking with (B) antibody 101F, (C) antibody D25, (D) antibody AM22, (E) antibody palivizumab, or (F) antibody motavizumab.

mRNA expression levels of proinflammatory cytokines IL-1β (Figure S7D), tumor necrosis factor α (TNF-α) (Figure S7E) and IL-6 (Figure S7F) were measured by RT-qPCR.

Q74 truncated monomer-induced immunoreactivity focuses on RSV F antigenic site II and site Ø

To investigate the immunofocusing neutralization mechanisms of Q74 truncated monomer, a biolayer interferometry (BLI)-based competition binding assay was carried out between RSV antigenic site antibodies and sera from RSV immunogen-immunized mice. First, the HIS1K biosensor was used to capture the DS-Cav1 immunogen. Second, RSV antigenic site antibodies and the immunized sera were added sequentially, and the binding ability of the immunized sera in the presence of saturating concentrations of the antigenic site antibody was measured (Figure 6A).

The inability of mAb 101F (site IV) to block binding of the immunized sera to the antigen indicated that the immunized sera had a very low percentage or no antibodies directed against antigenic site IV (Figure 6B). When mAb D25 was immobilized, the binding of Q74 truncated monomer-immunized sera to the antigen was blocked mildly, suggesting that the immunized sera have a higher percentage

of antibodies directed against antigenic site Ø (Figure 6C). The immobilization of mAb AM22 of the same antigenic site Ø exhibited an identical tendency (Figure 6D). When mAb palivizumab was immobilized, the binding of Q74 truncated monomer-immunized sera to the antigen was completely blocked, suggesting that most antibodies in immunized sera targeted antigenic site II (Figure 6E). The immobilization of mAb motavizumab of the same antigenic site II exhibited an identical tendency (Figure 6F). In contrast, the immunized sera of Q74 full-length trimer and DS-Cav1 contain complex polyclonal antibodies directed against multiple antigenic sites and are unable to be blocked from binding to the antigen by a single antibody. To sum up, the immunoreaction elicited by the Q74 truncated monomer concentrated on RSV F antigenic site II and site Ø.

DISCUSSION

We have found that RSV F protein monomer designed based on immunofocusing can induce immunogenicity and resistance against RSV infection comparable to DS-Cav1. The immunofocused Q74 truncated monomer elicits comparable neutralizing antibodies and protection to DS-Cav1, while demonstrating superior epitope focusing capability and structural stability. The Q74 truncated monomer is a truncated protein designed to include the head and neck regions of the RSV F protein. The termination site of its F1 protein is at the 336th aa, and the T72C-V76C disulfide bond mutation and K87L cavity-filling mutation are introduced, thus enabling the Q74 truncated monomer to stably maintain the PreF conformation. Our results show that the Q74 truncated monomer retains antigenic site Ø and II but lacks antigenic site IV and I. Therefore, the Q74 truncated monomer not only induces neutralizing and binding antibodies comparable to DS-Cav1 but also focuses the immune response on PreF (antigenic site Ø and II), resulting in immunofocusing and immunoprotection.

Comparative analysis of protein denaturation parameters revealed that the Q74 truncated monomer exhibited the highest onset temperature (62°C) and maximal Tm1 value among the tested immunogens. These results demonstrate superior structural stability of the Q74 truncated monomer relative to other trimeric proteins. In addition, the binding ability of Q74 truncated monomer to mAb did not change significantly after 1, 3, and 5 rounds of freeze-thaw, while the binding ability of trimeric protein to mAb almost disappeared after 3 and 5 rounds of freeze-thaw. These stability metrics collectively establish the Q74 monomer's superior structural robustness, strongly supporting its potential as a next-generation vaccine candidate.

In previous studies, the vaccine antigen designs of GSK, Pfizer, Moderna, and Janssen focused on stabilizing the PreF protein trimer conformation, such as DS-Cav1[12] (with mutations S155C-S290C, S190F, and V207L), 847[19] (with T103C-I148C, S190I, and D486S), SC-TM[15] (with N67I, S215P, and E487Q), TriM-5[16] (with N227M, L203F, and S509F), and so on. Our research, however, took a different approach to answer and verify that the RSV F protein monomer designed based on immunofocusing can induce immune protection. Previous studies[29] on the monomeric uncleaved RSV F antigen demonstrated that Δp27 furin$_{mut}$ F$_{ecto}$ contains a 23-residue deletion in the p27 fragment and point mutations at the furin cleavage sites, which prevent intracellular cleavage by furin. Characterization of Δp27 furin$_{mut}$ F$_{ecto}$ revealed that it exists predominantly as a monomeric state as determined by size-exclusion chromatography (SEC) coupled with multi-angle light scattering analysis. Furthermore, surface plasmon resonance and SEC analyses confirmed the binding capability of Δp27 furin$_{mut}$ F$_{ecto}$ to D25, motavizumab, and 101F. These findings suggest that the RSV F protein monomer retains PreF-specific neutralizing epitopes, thereby establishing a fundamental basis for our current research. Moreover, our results also demonstrate that the Q74 truncated monomer can induce a relatively high neutralizing titer (GMT = 439), thus confirming the feasibility of the immunofocusing monomer.

However, a previous study showed that although the monomeric head-only RSV F immunogen i-273 has good thermal stability, it fails to effectively induce neutralizing antibodies against RSV.[30] There are 4 possible reasons for this. (1)There is a difference in the aa lengths between i-273 and the Q74 truncated monomer. The F1 protein of the Q74 truncated monomer has 30 more aa than that of i-273, which may result in the Q74 truncated monomer containing more antigenic sites that can stimulate immune responses. (2) In the Q74 truncated monomer, the arrangement of F2 and F1 is the same as that of the native RSV F protein, while in i-273, RSV F1 is positioned before F2. This difference in arrangement might change the topological structure of F protein, thus leading to its inability to induce effective neutralizing antibodies. (3) The T72C-V76C disulfide bond mutation and K87L cavity-filling mutation, which were found in our previous studies to stabilize the PreF conformation, have been introduced into the Q74 truncated monomer, enabling the truncated monomer to be stabilized in the PreF conformation. In contrast, the i-273 antigen, which already includes the 4 mutations found in DS-Cav1, harbors an additional 13 mutations. The excessive number of mutations might have altered its immunogenicity. (4) The i-273 has a weak affinity for the mAb motavizumab, while the Q74 truncated monomer we designed has a strong binding ability to mAb motavizumab and palivizumab. The difference in binding ability to antibodies indicates that there are differences in antigenic site II, which may be the reason why Q74 can induce relatively high levels of neutralizing antibodies.

Additionally, our research has certain limitations. First, although we have conducted structural prediction, protein peptide mapping analysis, and protein antigenicity characterization, we lack direct structural data of the Q74 truncated monomer and cannot determine whether the Q74 truncated monomer is stabilized in PreF conformation. Second, our immunization and challenge studies were limited to a mouse model,[31] and we plan to conduct cotton rat challenge and non-human primate immunization in the next step. Finally, the molecular weight of the Q74 truncated monomer is small. Although it can successfully induce neutralizing antibodies against

RSV A and B strains, there is still room for improvement in its immunogenicity. Currently, we are carrying out the preparation of nanoparticle vaccines based on the Q74 truncated monomer immunogen, expecting to enhance its immunogenicity and immunoprotection through antigen enrichment.[32]

In conclusion, we obtain a truncated RSV F protein monomer immunogen based on immunofocusing. This finding may break the common perception that only the RSV PreF trimeric protein can induce strong immune protection. Our research provides a different strategy for the RSV vaccine design.

MATERIALS AND METHODS

Design of RSV F immunogens

To design more stable RSV F immunogens in the PreF conformation, we initially removed the P27 fragment and the furin-like protease cleavage site from the RSV F sequence. Then, we linked the F1 and F2 fragments with the GGSGGSGGS linker, converting the RSV F protein sequence into a single polypeptide chain. To focus the immune response to RSV's head and neck regions, we designed the Q74 truncated monomer immunogen by truncated the F1 fragment at aa 336 and added T72C-V76C disulfide bond mutation and K87L cavity-filling mutation, which had been found in previous experiments to stabilize the PreF conformation. Simultaneously, the Q74 full-length trimer immunogen, with T72C-V76C and K399C-S485C disulfide bond mutations, K87L cavity-filling mutation, and a T4-foldon trimerization domain attached at F1 aa 513, was designed to compare its immunogenicity to that of the DS-Cav1 immunogen. DS-Cav1 was used as a comparison immunogen in our study.[12]

Predictive structure modeling of RSV F immunogens

The structures of RSV PreF protein (PDB: 7LUC) and PostF protein (PDB: 3RRT) were obtained from the RCSB database. The sequences of mAb 101F (PDB: 3QQ9) and mAb D25 (PDB: 4JHA) were downloaded from the NCBI database. The interactions of Q74 truncated monomer, Q74 full-length trimer, and DS-Cav1 immunogens with mAb 101F and mAb D25 were predicted by the AlphaFold3 Server.[33] For ranking of the full complex, we used the pLDDT score and ranking_score (higher is better). The latter uses overall structure confidences (predicted template modeling and interface predicted template modeling). The predictive metrics for our final structure were comprehensively reported as follows: "fraction_disordered": 0.03, "has_clash": 0.0, "iptm": 0.74, "num_recycles": 10.0, "ptm": 0.75, and "ranking_score": 0.75. Additionally, we verified whether key antigenic regions align with experimentally resolved structures. Then, the best predicted results were selected and presented. Visual mapping was performed using the Pymol Molecular Graphics System (version 2.6.0). Schematic representation of protein sequences and mutations were mapped using IBS (http://ibs.biocuckoo.org/).

Cells and viruses

Expi293F cells (Thermo Fisher, catalog no. A14527) were maintained in OPM-293 CD05 Medium (OPM Biosciences, catalog no. 81075-001) at 37°C and 8% CO_2 at 125 rpm. HEp-2 cells (American Type Culture Collection [ATCC], catalog no. CCL-23) were grown in DMEM medium (Hyclone, catalog no. SH30243.01) containing 10% fetal bovine serum (TransGen Biotech, catalog no. FS301-02) and 1% penicillin/streptomycin (Gibco, catalog no. 15070063) at 37°C and 5% CO_2. RSV strain A2 (ATCC, catalog no. VR-1540P) and strain B18537 (ATCC, catalog no. VR-1580) were purchased from ATCC. RSV virus amplification, titration, and neutralizing antibody detection assays were performed using HEp-2 cells.

Expression, purification and characterization of RSV F immunogens and antibodies

The RSV F immunogens and mAbs were constructed on the pcDNA3.1 vector and expressed in Expi293F cells following transfection. After 5–7 days of plasmid transfection, cell culture supernatants were collected. A 2-step chromatography was performed sequentially using an anion chromatography column followed by a cation chromatography column. The protein eluate obtained from these steps was then concentrated by ultrafiltration for subsequent protein quantification and characterization experiments. The purified RSV F immunogens and mAbs were reduced, denatured, and separated on a 10% SDS-PAGE gel. After that, the proteins were electro-transferred to a polyvinylidene fluoride membrane. Blocking was then performed, and membranes were first incubated with mAb D25 and mAb motavizumab at 4°C overnight. Afterward, membranes were washed and incubated with anti-human IgG-HRP secondary antibody at 25°C for 1 h. Signals were detected and visualized using the ChemiDoc MP imaging system (Bio-Rad).

Antigenic characterization of RSV F immunogens

Indirect ELISA: the purified Q74 full-length trimer, Q74 truncated monomer, and DS-Cav1 immunogens were serially triple diluted from a starting 100 ng/well. The diluted proteins were immobilized in duplicate for each sample on a 96-well plate (Corning, catalog no. 9018) and incubated at 4°C overnight. Subsequently, after blocking with 1% BSA, 100 μL of site Ø antibody D25, site Ø antibody AM22, site II antibody palivizumab, site II antibody motavizumab, site IV antibody 101F and site I antibody 4D7 at a concentration of 2 μg/mL were added and incubated at 37°C for 1 h. Finally, anti-human IgG-HRP, diluted 1:10,000, was used for detection. The mean ± SD of the samples was calculated to evaluate their binding activity to specific mAbs. The binding abilities of DS-Cav1 were detected for comparison.

Sandwich ELISA: RSV-specific mAb motavizumab/D25-HRP pair and mAb motavizumab/101F-HRP pair were used to detect the binding activity of Q74 full-length trimer and Q74 truncated monomer. The RSV mAb motavizumab at a concentration of 2 μg/mL were immobilized on a 96-well plate (Corning, catalog no. 9018) and incubated at 4°C overnight. Subsequently, after blocking with 1% BSA, the purified Q74 full-length trimer, the Q74 truncated monomer, and DS-Cav1 immunogens with 2-fold serial dilution from a starting 50 ng/well were added and incubated at 37°C for 1 h. Finally, mAb D25-HRP and 101F-HRP at a concentration of

4 μg/mL, were used for detection. The mean ± SD of the samples was calculated to evaluate their binding activity. The binding abilities of DS-Cav1 were detected for comparison.

Assessments of physical stability

First, the purified Q74 full-length trimer, Q74 truncated monomer, and DS-Cav1 immunogens underwent high-temperature treatment (at 50°C, 60°C, and 70°C for 1 h each) and freeze-thaw cycles (1, 3, and 5 rounds). Second, the immunogens were diluted with PBS to 50 μg/mL for sandwich ELISA using mAb motavizumab/D25-HRP and mAb motavizumab/101F-HRP. After that, the antibody binding capacities after treatment under different conditions were calculated and compared with those before being treated to assess the effect of different treatments on protein conformation. A ratio closer to 1 indicates a smaller change, suggesting greater protein conformational stability.

Measurement of Tm values

The assay was carried out in PBS buffer using 1 mg/mL purified Q74 full-length trimer, Q74 truncated monomer, and DS-Cav1 immunogens. The samples were loaded into capillaries (NanoTemper Technologies, catalog no. PR-C006) and run in the Prometheus NT.48 nanoDSF instrument (NanoTemper Technologies). Immunogen unfolding was monitored during heating at 1°C/min from 20°C to 95°C with 60% excitation power. The changes in the 350:330 nm tryptophan fluorescence ratio during protein denaturation were recorded, and its first derivative was used to calculate the Tm value. The 350:330 nm fluorescence ratio and its first derivative were calculated with the PR.StabilityAnalysis software. Three individual measurements were performed, and the average value of Tm value is shown.

Mouse immunizations and challenges

Female 6- to 8-week-old specific pathogen-free (SPF)-grade BALB/c mice were obtained from the Institute of Medical Biology, Chinese Academy of Medical Sciences (IMBCAMss), and housed in an SPF-grade animal facility (mouse license no. SCXK [Dian] K2019-0002). The experimental protocol received formal approval from the IMBCAMs Animal Ethics Committee (Approval No.: DWSP02306048). First, at week 0, all the mice were infected intranasally with 5×10^5 PFU RSV A2 virus to create an RSV infection background similar to that seen in the elderly. Second, at week 4, the mice were immunized intramuscularly with 10 μg of either Q74 truncated monomer, Q74 full-length trimer, or DS-Cav1 per mouse, using 50 μg aluminum hydroxide as an adjuvant. The viral infection group received a second RSV A2 virus infection. At week 7, some mice were challenged by intranasal infection with 5×10^5 PFU RSV B18537 virus. At week 10, other mice were challenged by intranasal infection with 5×10^5 PFU RSV A2 virus. Afterward, the body weights of the virus-infected mice were continuously monitored. On day 4 post-infection, the mice were euthanized, and lung tissues were collected for virus load determination. In addition, sera were acquired via the inner-eye canthus at weeks 4, 7, and 10, respectively, for neutralizing antibody and binding antibody detection.

Sera binding analysis

The IgG antibody titers against the RSV PreF and PostF immunogens in mouse sera were detected by the indirect ELISA. Initially, mice sera were diluted 200-fold and then serial 2-fold dilutions were conducted. Meanwhile, the negative control sera were equally mixed and also diluted 200-fold. Subsequently, 100 μL of the diluted sera per well was added to the RSV PreF or PostF immunogen-coated plate and incubated at room temperature for 1 h. Then, 100 μL/well of the goat anti-mouse IgG-HRP antibody diluted 1:10,000 was added and incubated at room temperature for another 1 h. Next, 100 μL per well of the TMB substrate solution was added and incubated at room temperature for 5–10 min. Finally, 50 μL/well of the stop solution was added to terminate the reaction, and the plate was read on an absorbance plate reader at 450 nm (reference at 630 nm). The cutoff value was established as 2.1 times the OD value of the negative control group. The antibody titer was defined as the dilution factor that was equal to or greater than the cutoff value. The GMT of the sera of each group was then calculated.

RSV live-virus neutralization assays

RSV-specific neutralizing antibody detection is essential for the evaluation of the immunization effect of the RSV vaccine. In this method, the RSV cytopathic effect (CPE) neutralization test was established by using HEp-2 cells with the RSV A2 strain and the RSV B18537 strain. Briefly, RSV was incubated with serially diluted test sera (with an initial dilution of 50-fold, followed by 4-fold dilutions) in a 96-well plate at 37°C for 1 h. Subsequently, 10,000 HEp-2 cells were added to each well. After incubation at 37°C in a 5% CO_2 environment for 4–7 days, the crystal violet solution was added and incubated for 30 min. Then, the CPE was observed and recorded. The number of CPE^+ and CPE^- wells was accumulated. The 50% inhibitory dilution (ID_{50}) was regarded as the serum dilution where the CPE was diminished by 50% relative to the virus control wells. The neutralizing antibody titer (ID_{50}) was calculated using the Reed-Muench method.

BALF IgA detection

Mice were sacrificed by cervical dislocation, and the chest cavity was opened to expose the trachea. A small incision was made in the trachea, and a cannula was inserted. Sterile PBS (0.5 mL) was instilled and aspirated via a syringe attached to the cannula; this lavage procedure was repeated twice. The collected fluid was pooled into a 1.5-mL microcentrifuge tube, centrifuged at $300 \times g$ for 5 min, and the supernatant was stored at −20°C. For ELISA, microtiter plates (Corning, catalog no. 9018) were coated overnight at 4°C with RSV DS-CaV1 protein (5 μg/mL, 100 μL/well). The next day, the plates were washed 3 times with PBS with Tween 20 (PBST) and blocked with 1% BSA at 37°C for 1 h. Diluted BALF samples (100 μL/well) were added and incubated at 37°C for 2 h. After 5 PBST washes, HRP-conjugated anti-mouse IgA antibody (SouthernBiotech, catalog no. 1040-05) was added and incubated at 37°C for 1 h. Following 5 PBST washes, TMB substrate (Solarbio, catalog no. PR1200) was added for color

development, and the reaction was stopped with 2 M H_2SO_4. Absorbance was measured at 450 nm (reference 630 nm) using a microplate reader.

Cellular immunity assays

Following euthanasia by decapitation and necropsy, spleens were aseptically removed from the mice. Splenic tissue was gently dissociated through a 70-μm cell strainer (Falcon, catalog no. 352350) into RPMI 1640 medium (Gibco, catalog no. C11875500BT). The resulting cell suspension was layered over 3 mL mouse lymphocyte separation medium (Dakewe, catalog no. 7211011) in a 15-mL centrifuge tube and centrifuged at 800 × *g* for 30 min. The lymphocyte layer was collected, washed twice with RPMI 1640 medium, and resuspended. After cell counting, the lymphocyte concentration was adjusted to 1×10^7 cells/mL in RPMI 1640 medium for subsequent assays.

Cytokine secretion in stimulated splenic lymphocytes supernatants was quantified by ELISA. Splenic lymphocytes were seeded at 1×10^6 cells/well (100 μL/well) in 96-well plates, followed by the addition of immunostimulants (DS-Cav1, 10 μg/mL). After 24 h incubation at 37°C, supernatants were collected for analysis. For ELISA, coating: plates were coated overnight at 4°C with PBS-diluted capture antibodies (anti-IL-4, anti-IL-2, and anti-IFN-γ; 4 μg/mL); blocking: 1 h at 37°C with 1% BSA/PBS; sample incubation: 3 h at room temperature; detection: biotin-conjugated detection antibodies (anti-IL-4, anti-IL-2, and anti-IFN-γ; 2 μg/mL in 1% BSA; 1 h) and HRP-streptavidin (1 μg/mL; 30 min); signal development: TMB substrate addition and reaction termination with 2 M H_2SO_4; and reading: absorbance at 450 nm (SYNERGY 4 microplate reader, BioTek).

Splenic lymphocytes were analyzed for antigen-specific cytokine secretion using ELISpot. Pre-assembled MabTech antibody-coated strips (IFN-γ: 3321-4AST-10, IL-4: 3311-4APW-2, and IL-2: 3441-4APW-2) were washed 4× with sterile PBS (200 μL/well) and blocked with 10% serum-contained complete medium (Dakewe, catalog no. 6015012, 200 μL/well, 30 min, room temperature). Splenic lymphocytes (5×10^5 cells/well in 100 μL RPMI 1640) and RSV-F protein stimulant (2 μg/well) were added to designated wells. Plates were incubated 12–48 h (37°C, 5% CO_2). Cells were removed by plate inversion, followed by 5× PBS washes (200 μL/well). Detection antibody (1 μg/mL in PBS-0.5% fetal calf serum [FCS], 100 μL/well) was incubated for 2 h (room temperature), washed, then streptavidin-ALP (1:1,000 in PBS-0.5% FCS, 100 μL/well) was added for 1 h (room temperature). After the final washes, spots were developed with filtered BCIP/NPT-plus substrate (100 μL/well; color development monitored visually). Reactions were terminated by water rinsing. Dried membranes were analyzed using an automated ELISpot reader (CTL).

Antigenic site antibodies competition analysis of immunized sera by BLI

All BLI experiments were performed on an Octet R4 instrument (Sartorius). DS-Cav1 was diluted to 20 μg/mL in a phosphate buffer containing 2% BSA and 0.02% Tween 20. After loading the DS-Cav1 antigen onto the HIS1K sensor for 120 s, a baseline signal was established in the buffer for 300 s. The sensor containing RSV DS-Cav1 was then immersed in purified RSV F antibodies (101F, D25, AM22, palivizumab, and motavizumab) for 900 s to block the RSV F-associated antigenic site. After completing sufficient blocking, it was continuously exposed to immunized mice sera (equal amounts of serum from 5 mice in each group were mixed) for 540 s. To prevent the dissociation of blocking antibodies caused by the addition of serum, an equivalent amount of antibodies was added to the buffer to ensure that antigen-related epitopes remained blocked. Using the data analysis software, we calculated the reduction in the binding signal of the sera in the presence of the RSV F mAbs.

Lung tissue viral load and proinflammatory cytokines

To determine the RSV viral load, lung tissues were first ground, after which viral RNA was extracted using the Viral RNA Extraction Kit (QIAGEN, catalog no. 52906). After that, cDNA was synthesized with PrimeScript RT Master Mix (Takara, catalog no. RR036A). Subsequently, RT-qPCR was carried out using AceQ Universal U + Probe Master Mix (Vazyme, catalog no. Q513) to quantify the copy of the RSV N gene and proinflammatory cytokines.

A standard curve was then plotted using the RSV N protein plasmid, and the viral load was calculated as the copies per milligram of lung tissue. qPCR targeting the RSV A2 strain N gene employed the following primers and probe: forward primer (5′-GGCAGTAG AGTTGAAGGGATTTC-3′), reverse primer (5′-TGCACACTAGC ATGTCCTAAC-3′), and the TaqMan probe (5′-FAM-TATGAAT GCCTATGGTGCAGGGCA-BHQ1-3′). qPCR targeting the RSV 1580 strain gene employed the following primers and probe: forward primer (5′-CCTATGGTTCAGGGCAAGTAAT-3′), reverse primer (5′-CTTCCACAACTTGCTCCATTTC-3′), and the TaqMan probe (5′-FAM-CTAGGTCATGCTAGTGTCCAGGCA-BHQ1-3′).

To measure proinflammatory cytokine gene expression by relative RT-qPCR, cell samples using ChamQ Universal SYBR qPCR Master Mix (Vazyme, catalog no. Q711-02) were used according to the manufacturer's instructions. Primer pairs specific for each target inflammatory cytokine (e.g., TNF-α, IL-6, IL-1β) and a stable housekeeping gene (glyceraldehyde 3-phosphate dehydrogenase) were purchased. Each Ct (threshold cycle) value for both target and housekeeping genes was recorded. Inflammatory cytokine expression levels in each experimental group were reported as mean ± SD of $2^{-\Delta\Delta Ct}$ values, normalizing all data to the chosen calibrator. This ΔΔCt method yielded a relative quantification of cytokine mRNA expression compared to the control group.

Histopathologic assays and immunohistochemical analysis of CD68⁺ macrophages

The mice lung tissues were embedded in paraffin after fixation in 4% paraformaldehyde and sectioned for histological analysis. Following the processes of dewaxing and hydration, the sections were subjected to staining with hematoxylin and 25% eosin for hematoxylin and

eosin staining. Images were captured using a panoramic slice-scanner system (3D HISTECH), and basic pathological changes were determined. A 4-tiered histopathological grading system was used to assess tissue alterations, with scoring criteria as follows. grade 0: normal tissue architecture; grade 1: minimal pathological changes; grade 2: moderate lesions (not yet severe); grade 3: marked pathological alterations; grade 4: severe lesions involving the entire tissue.

Immunohistochemical (IHC) staining was performed by ServiceBio according to standard protocols. IHC analysis of $CD68^{+}$ macrophages was performed using Anti-CD68 Rabbit polyclonal antibody (ServiceBio, catalog no. GB113109-50). IHC analysis was performed using AIpathwell software to quantify staining intensity and the proportion of positive cells. CD68 expression levels were semi-quantitatively assessed using the H-score, calculated as $\sum(\text{pi} \times \text{i})$, where pi represents the percentage of positive cells and *i* denotes staining intensity (0 = negative; 1 = weak; 2 = moderate; 3 = strong). H-scores range from 0 to 300, with higher values indicating greater CD68 expression.

Statistical analysis

Statistical comparisons were performed using 1-way analysis of variance (ANOVA) between different groups; * $p < 0.05$; ** $p < 0.01$; *** $p < 0.001$; ****$p < 0.0001$; ns, not significant.

DATA AVAILABILITY

All data are available in the main text or the supplemental information.

ACKNOWLEDGMENTS

This work was supported by the Chinese Academy of Medical Sciences Innovation Fund for Medical Sciences (grant no. 2022-I2M-3-001), the Youth Program of National Natural Science Foundation of China (grant no. 82402138), the General Program of National Natural Science Foundation of China (grant no. 82372225), the National Key R&D Program of China (grant no. 2023YFC2307900), the Science and Technology Leading Talent Program of Yunnan Province (grant no. 202405AB350002), the Xindian Talent Support Program Young Talents in Yunnan Province, Chinese Academy of Medical Sciences Innovation Fund for Medical Sciences (grant no. 2023-I2M-2-001).

AUTHOR CONTRIBUTIONS

Q.L. and Y.W. conceived and supervised the study. Q.L., J.C., R.B., and Z.L. developed the methodology. Q.L., Z.L., J.C., Q.H., H.X., H.L., H.Z., Y.Y., R.B., S.X., Y.B., B.L., L.H., M.Z., J.T., HW.L., R.L., and J.D. conducted the investigation. Q.L. and Z.L. performed the visualization. Q.L., J.C., H.L., R.B., Z.L., and Y.W. wrote the original draft. Q.L. and Y.W. reviewed and edited the manuscript. All authors reviewed and approved the final manuscript.

DECLARATION OF INTERESTS

The authors declare no competing interests.

SUPPLEMENTAL INFORMATION

Supplemental information can be found online at https://doi.org/10.1016/j.ymthe.2025.07.040.

REFERENCES

1. Vandini, S., Biagi, C., and Lanari, M. (2017). Respiratory Syncytial Virus: The Influence of Serotype and Genotype Variability on Clinical Course of Infection. Int. J. Mol. Sci. *18*, 1717. https://doi.org/10.3390/ijms18081717.
2. Colosia, A., Costello, J., McQuarrie, K., Kato, K., and Bertzos, K. (2023). Systematic literature review of the signs and symptoms of respiratory syncytial virus. Influenza Other Respir. Viruses *17*, e13100. https://doi.org/10.1111/irv.13100.
3. Li, Y., Wang, X., Blau, D.M., Caballero, M.T., Feikin, D.R., Gill, C.J., Madhi, S.A., Omer, S.B., Simões, E.A.F., Campbell, H., et al. (2022). Global, regional, and national disease burden estimates of acute lower respiratory infections due to respiratory syncytial virus in children younger than 5 years in 2019: a systematic analysis. Lancet *399*, 2047–2064. https://doi.org/10.1016/S0140-6736(22)00478-0.
4. Nam, H.H., and Ison, M.G. (2019). Respiratory syncytial virus infection in adults. BMJ *366*, l5021. https://doi.org/10.1136/bmj.l5021.
5. Mazur, N.I., Terstappen, J., Baral, R., Bardají, A., Beutels, P., Buchholz, U.J., Cohen, C., Crowe, J.E., Jr., Cutland, C.L., Eckert, L., et al. (2023). Respiratory syncytial virus prevention within reach: the vaccine and monoclonal antibody landscape. Lancet Infect. Dis. *23*, e2–e21. https://doi.org/10.1016/S1473-3099(22)00291-2.
6. Chen, X., Xu, B., Guo, J., Li, C., An, S., Zhou, Y., Chen, A., Deng, L., Fu, Z., Zhu, Y., et al. (2018). Genetic variations in the fusion protein of respiratory syncytial virus isolated from children hospitalized with community-acquired pneumonia in China. Sci. Rep. *8*, 4491. https://doi.org/10.1038/s41598-018-22826-4.
7. McLellan, J.S., Chen, M., Leung, S., Graepel, K.W., Du, X., Yang, Y., Zhou, T., Baxa, U., Yasuda, E., Beaumont, T., et al. (2013). Structure of RSV fusion glycoprotein trimer bound to a prefusion-specific neutralizing antibody. Science *340*, 1113–1117. https://doi.org/10.1126/science.1234914.
8. McLellan, J.S., Yang, Y., Graham, B.S., and Kwong, P.D. (2011). Structure of respiratory syncytial virus fusion glycoprotein in the postfusion conformation reveals preservation of neutralizing epitopes. J. Virol. *85*, 7788–7796. https://doi.org/10.1128/JVI.00555-11.
9. Ananworanich, J., and Heaton, P.M. (2021). Bringing Preventive RSV Monoclonal Antibodies to Infants in Low- and Middle-Income Countries: Challenges and Opportunities. Vaccines (Basel) *9*, 961. https://doi.org/10.3390/vaccines9090961.
10. Chang, L.A., Phung, E., Crank, M.C., Morabito, K.M., Villafana, T., Dubovsky, F., Falloon, J., Esser, M.T., Lin, B.C., Chen, G.L., et al. (2022). A prefusion-stabilized RSV F subunit vaccine elicits B cell responses with greater breadth and potency than a postfusion F vaccine. Sci. Transl. Med. *14*, eade0424. https://doi.org/10.1126/scitranslmed.ade0424.
11. Graham, B.S., Gilman, M.S.A., and McLellan, J.S. (2019). Structure-Based Vaccine Antigen Design. Annu. Rev. Med. *70*, 91–104. https://doi.org/10.1146/annurev-med-121217-094234.
12. McLellan, J.S., Chen, M., Joyce, M.G., Sastry, M., Stewart-Jones, G.B.E., Yang, Y., Zhang, B., Chen, L., Srivatsan, S., Zheng, A., et al. (2013). Structure-based design of a fusion glycoprotein vaccine for respiratory syncytial virus. Science *342*, 592–598. https://doi.org/10.1126/science.1243283.
13. Gidwani, S.V., Brahmbhatt, D., Zomback, A., Bassie, M., Martinez, J., Zhuang, J., Schulze, J., McLellan, J.S., Mariani, R., Alff, P., et al. (2024). Engineered dityrosine-bonding of the RSV prefusion F protein imparts stability and potency advantages. Nat. Commun. *15*, 2202. https://doi.org/10.1038/s41467-024-46295-8.
14. Sanders, R.W., and Moore, J.P. (2021). Virus vaccines: proteins prefer prolines. Cell Host Microbe *29*, 327–333. https://doi.org/10.1016/j.chom.2021.02.002.
15. Krarup, A., Truan, D., Furmanova-Hollenstein, P., Bogaert, L., Bouchier, P., Bisschop, I.J.M., Widjojoatmodjo, M.N., Zahn, R., Schuitemaker, H., McLellan, J.S., and Langedijk, J.P.M. (2015). A highly stable prefusion RSV F vaccine derived from structural analysis of the fusion mechanism. Nat. Commun. *6*, 8143. https://doi.org/10.1038/ncomms9143.
16. Liang, Y., Shao, S., Li, X.Y., Zhao, Z.X., Liu, N., Liu, Z.M., Shen, F.J., Zhang, H., Hou, J.W., Zhang, X.F., et al. (2024). Mutating a flexible region of the RSV F protein can stabilize the prefusion conformation. Science *385*, 1484–1491. https://doi.org/10.1126/science.adp2362.
17. Stewart-Jones, G.B.E., Thomas, P.V., Chen, M., Druz, A., Joyce, M.G., Kong, W.P., Sastry, M., Soto, C., Yang, Y., Zhang, B., et al. (2015). A Cysteine Zipper Stabilizes a Pre-Fusion F Glycoprotein Vaccine for Respiratory Syncytial Virus. PLoS One *10*, e0128779. https://doi.org/10.1371/journal.pone.0128779.
18. Crank, M.C., Ruckwardt, T.J., Chen, M., Morabito, K.M., Phung, E., Costner, P.J., Holman, L.A., Hickman, S.P., Berkowitz, N.M., Gordon, I.J., et al. (2019). A proof

of concept for structure-based vaccine design targeting RSV in humans. Science *365*, 505–509. https://doi.org/10.1126/science.aav9033.

19. Che, Y., Gribenko, A.V., Song, X., Handke, L.D., Efferen, K.S., Tompkins, K., Kodali, S., Nunez, L., Prasad, A.K., Phelan, L.M., et al. (2023). Rational design of a highly immunogenic prefusion-stabilized F glycoprotein antigen for a respiratory syncytial virus vaccine. Sci. Transl. Med. *15*, eade6422. https://doi.org/10.1126/scitranslmed.ade6422.
20. Britton, A., Roper, L.E., Kotton, C.N., Hutton, D.W., Fleming-Dutra, K.E., Godfrey, M., Ortega-Sanchez, I.R., Broder, K.R., Talbot, H.K., Long, S.S., et al. (2024). Use of Respiratory Syncytial Virus Vaccines in Adults Aged >/=60 Years: Updated Recommendations of the Advisory Committee on Immunization Practices - United States, 2024. MMWR. Morb. Mortal. Wkly. Rep. *73*, 696–702. https://doi.org/10.15585/mmwr.mm7332e1.
21. Bakkers, M.J.G., Cox, F., Koornneef, A., Yu, X., van Overveld, D., Le, L., van den Hoogen, W., Vaneman, J., Thoma, A., Voorzaat, R., et al. (2024). A foldon-free prefusion F trimer vaccine for respiratory syncytial virus to reduce off-target immune responses. Nat. Microbiol. *9*, 3254–3267. https://doi.org/10.1038/s41564-024-01860-1.
22. Bruun, T.U.J., Do, J., Weidenbacher, P.A.B., Utz, A., and Kim, P.S. (2024). Engineering a SARS-CoV-2 Vaccine Targeting the Receptor-Binding Domain Cryptic-Face via Immunofocusing. ACS Cent. Sci. *10*, 1871–1884. https://doi.org/10.1021/acscentsci.4c00722.
23. Ma, C., Wang, L., Tao, X., Zhang, N., Yang, Y., Tseng, C.T.K., Li, F., Zhou, Y., Jiang, S., and Du, L. (2014). Searching for an ideal vaccine candidate among different MERS coronavirus receptor-binding fragments–the importance of immunofocusing in subunit vaccine design. Vaccine *32*, 6170–6176. https://doi.org/10.1016/j.vaccine.2014.08.086.
24. Xu, D., Powell, A.E., Utz, A., Sanyal, M., Do, J., Patten, J.J., Moliva, J.I., Sullivan, N.J., Davey, R.A., and Kim, P.S. (2024). Design of universal Ebola virus vaccine candidates via immunofocusing. Proc. Natl. Acad. Sci. USA *121*, e2316960121. https://doi.org/10.1073/pnas.2316960121.
25. Dosey, A., Ellis, D., Boyoglu-Barnum, S., Syeda, H., Saunders, M., Watson, M.J., Kraft, J.C., Pham, M.N., Guttman, M., Lee, K.K., et al. (2023). Combinatorial immune refocusing within the influenza hemagglutinin RBD improves cross-neutralizing antibody responses. Cell Rep. *42*, 113553. https://doi.org/10.1016/j.celrep.2023.113553.
26. Musunuri, S., Weidenbacher, P.A.B., and Kim, P.S. (2024). Bringing immunofocusing into focus. NPJ Vaccin. *9*, 11. https://doi.org/10.1038/s41541-023-00792-x.
27. Mashiyama, F., Hashimoto, K., Norito, S., Okabe, H., Sato, A., Kume, Y., Maeda, R., Sato, M., Sato, M., Kyozuka, H., et al. (2022). Neutralizing and Epitope-Specific Antibodies against Respiratory Syncytial Virus in Maternal and Cord Blood Paired Samples. Viruses *14*, 2702. https://doi.org/10.3390/v14122702.
28. Lin, M., Yin, Y., Zhao, X., Wang, C., Zhu, X., Zhan, L., Chen, L., Wang, S., Lin, X., Zhang, J., et al. (2025). A truncated pre-F protein mRNA vaccine elicits an enhanced immune response and protection against respiratory syncytial virus. Nat. Commun. *16*, 1386. https://doi.org/10.1038/s41467-025-56302-1.
29. Swanson, K.A., Balabanis, K., Xie, Y., Aggarwal, Y., Palomo, C., Mas, V., Metrick, C., Yang, H., Shaw, C.A., Melero, J.A., et al. (2014). A monomeric uncleaved respiratory syncytial virus F antigen retains prefusion-specific neutralizing epitopes. J. Virol. *88*, 11802–11810. https://doi.org/10.1128/JVI.01225-14.
30. Boyington, J.C., Joyce, M.G., Sastry, M., Stewart-Jones, G.B.E., Chen, M., Kong, W. P., Ngwuta, J.O., Thomas, P.V., Tsybovsky, Y., Yang, Y., et al. (2016). Structure-Based Design of Head-Only Fusion Glycoprotein Immunogens for Respiratory Syncytial Virus. PLoS One *11*, e0159709. https://doi.org/10.1371/journal.pone.0159709.
31. Zhang, G., Zhao, B., and Liu, J. (2024). The Development of Animal Models for Respiratory Syncytial Virus (RSV) Infection and Enhanced RSV Disease. Viruses *16*, 1701. https://doi.org/10.3390/v16111701.
32. Marcandalli, J., Fiala, B., Ols, S., Perotti, M., de van der Schueren, W., Snijder, J., Hodge, E., Benhaim, M., Ravichandran, R., Carter, L., et al. (2019). Induction of Potent Neutralizing Antibody Responses by a Designed Protein Nanoparticle Vaccine for Respiratory Syncytial Virus. Cell *176*, 1420–1431. https://doi.org/10.1016/j.cell.2019.01.046.
33. Abramson, J., Adler, J., Dunger, J., Evans, R., Green, T., Pritzel, A., Ronneberger, O., Willmore, L., Ballard, A.J., Bambrick, J., et al. (2024). Accurate structure prediction of biomolecular interactions with AlphaFold 3. Nature *630*, 493–500. https://doi.org/10.1038/s41586-024-07487-w.

YMTHE, Volume *33*

Supplemental Information

Immunofocusing design of a fusion glycoprotein monomer vaccine for respiratory syncytial virus

Qianqian Li, Zhiming Li, Jiamin Chen, Qiuju He, Hongjian Xiao, Haiwei Li, Huan Li, Heng Zhang, Yaoyun Yang, Rong Bi, Zichen Li, Song Xiao, Yanwei Bi, Bingyan Liang, Luxia Huang, Mengyi Zhang, Jincheng Tong, Haoyue Long, Ru Li, Jinmei Duan, Zhihua Li, and Youchun Wang

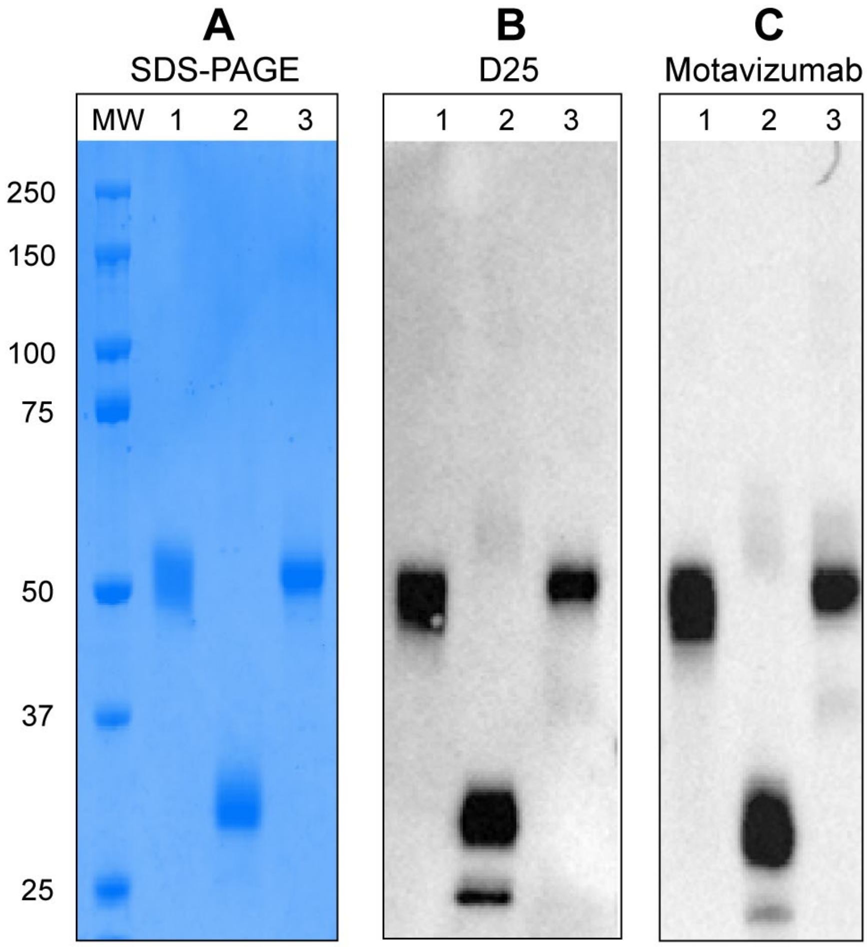

Figure S1. Characterization of RSV F immunogens, related to Figure 1. (A) Reduced SDS-PAGE analysis of purified RSV F immunogens. (B) Western blot analysis of purified RSV F immunogens using anti-RSV monoclonal antibody D25. (C) Western blot analysis of purified RSV F immunogens using anti-RSV monoclonal antibody Motavizumab. Lane1, Q74 trimer; lane2, Q74 monomer; lane3, DS-Cav1.

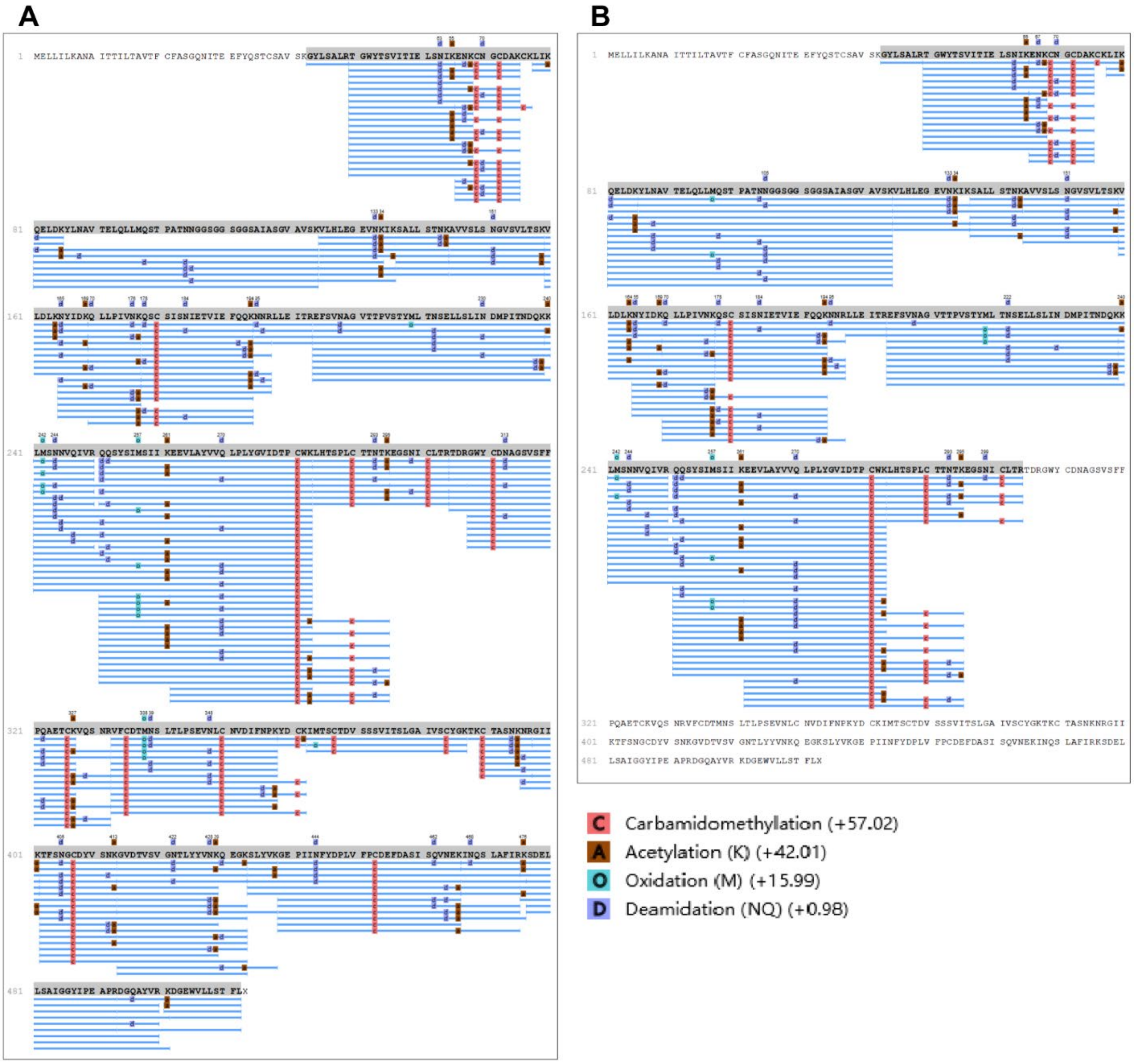

Figure S2. Peptide mapping analysis of RSV F immunogens, related to Figure 1. Sequence coverage of the Q74 trimer protein (A) and Q74 monomer protein (B) was calculated after identification of peptides detected in the peptide map. The amino acid sequence is shown with blue bars to indicate the identified regions. PTM was identified by searching the PEAKS database. The exact site of modification (carbamidomethylation, acetylation, oxidation and deamidation) were represented on the blue bars.

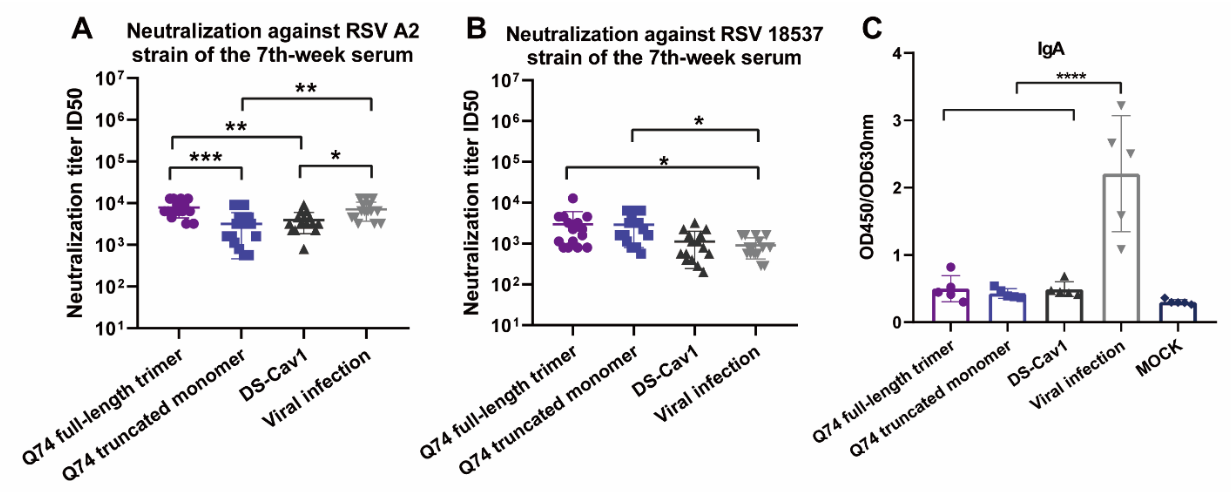

Figure S3. Evaluation of antibody response to RSV F immunogens in mice, related to Figure 4. Neutralizing antibody titers against RSV A2 (A) and B18537 (B) strains were assessed using a live virus neutralization assay in sera collected from mice (15 per group) at week 7 after immunization. Titers are reported as the 50% geometric mean titer (GMT) within each group as mean ± SD. GMTs were log10-transformed. (C) The fully immunized mice were euthanized 3 weeks after the 2ndboost immunization, and bronchoalveolar lavage fluid (BALF) was collected. Mucosal immunoreactivity in BALF samples was determined by assessing DS-CaV1-specific IgA. Statistical comparisons were performed using one-way analysis of variance (ANOVA) between different groups. * means $P < 0.05$; ** means $P < 0.01$; *** means $P < 0.001$; **** means $P < 0.0001$; ns means not significant.

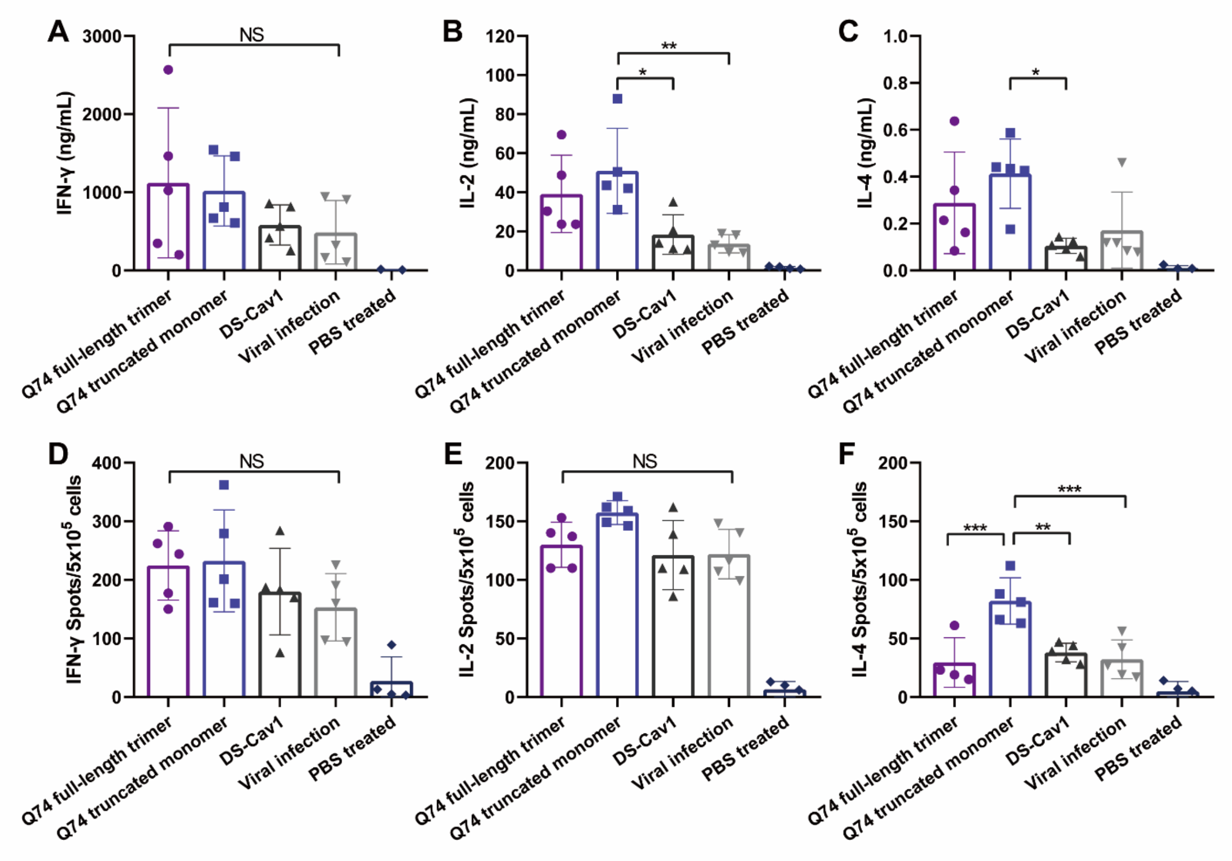

Figure S4. Evaluation of cellular immune response to RSV F immunogens in mice, related to Figure 4. The fully immunized mice were euthanized 3 weeks after the second booster immunization and splenic lymphocytes were collected. The levels of secreted IFN-γ (A), IL-2 (B) and IL-4 (C) in splenocytes after DS-CaV1 stimulation were assayed using ELISA. The numbers of IFN-γ- (D), IL-2- (E) and IL-4-secreting(F) splenocytes after DS-CaV1 stimulation were measured using Enzyme-linked immunospot assay (ELISpot). Statistical comparisons were performed using one-way analysis of variance (ANOVA) between different groups. * means $P < 0.05$; ** means $P < 0.01$; *** means $P < 0.001$; **** means $P < 0.0001$; ns means not significant.

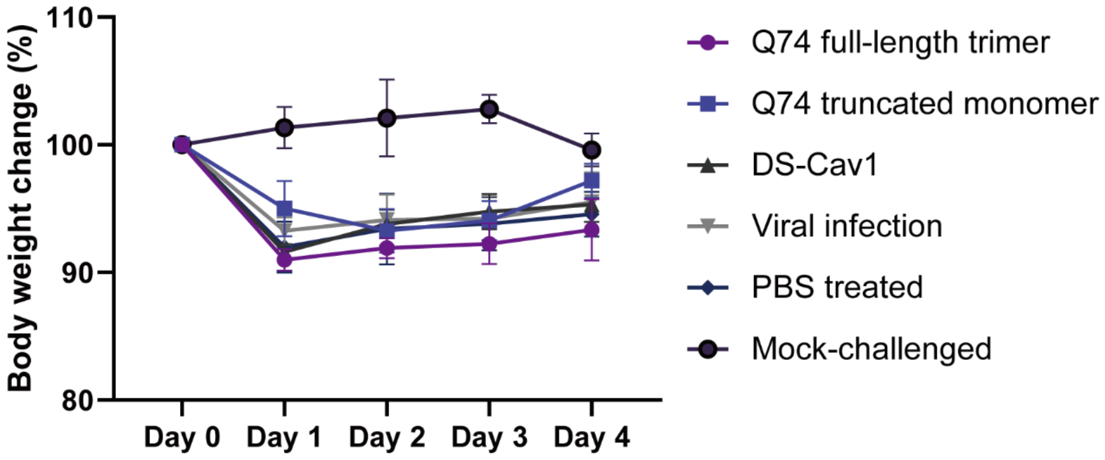

Figure S5. Body weight changes in mice following RSV infection, related to Figure 5. Daily body weight is shown as a percentage of the weight on day 0. PBS-treated: RSV-infected (positive control) group; Mock-challenged: Uninfected (negative control) group.

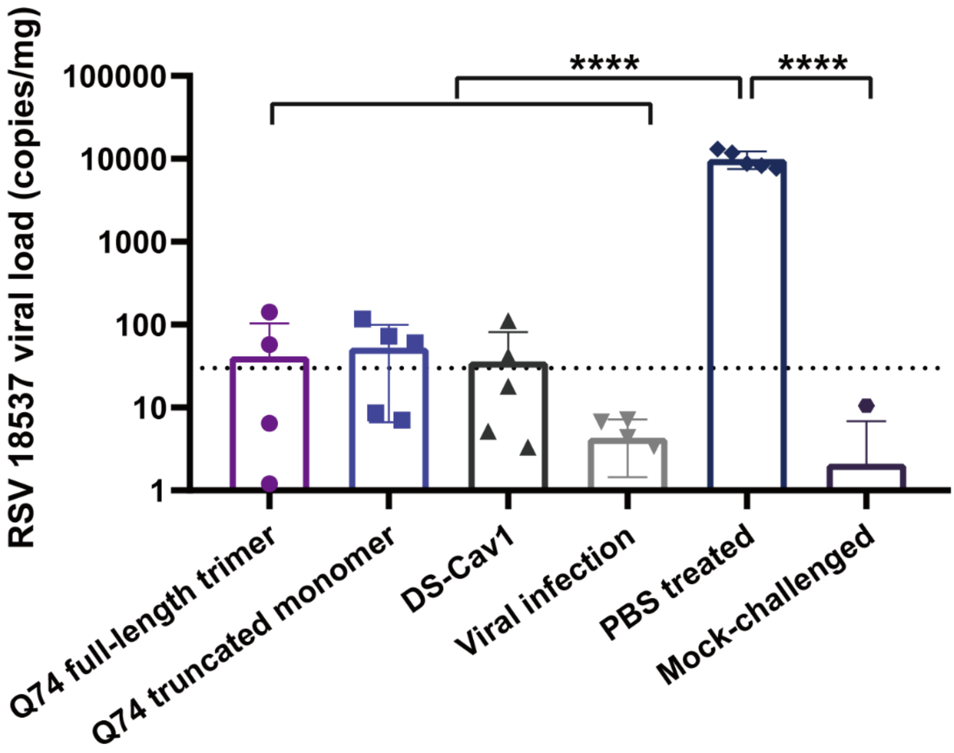

Figure S6. Protective efficacy of RSV F immunogens against RSV 18537 virus challenge., related to Figure 5. RSV viral load (RSV N gene copies) in the lung tissue after RSV 18537 virus challenge. Three weeks after immunization, mice were challenged with 5×10^5 PFU RSV 18537 virus at weeks 7. PBS treated animals served as viral infection model controls, mock-challenged animals served as non-viral infection model controls. Viral load was measured four days after challenge in the lung tissue via RT-qPCR and are reported as copies/mg within each group as mean ± SD. Statistical comparisons were performed using one-way analysis of variance (ANOVA) between different groups. * means $P < 0.05$; ** means $P < 0.01$; *** means $P < 0.001$; **** means $P < 0.0001$; LOD = limit of detection at 30 copies/mg.

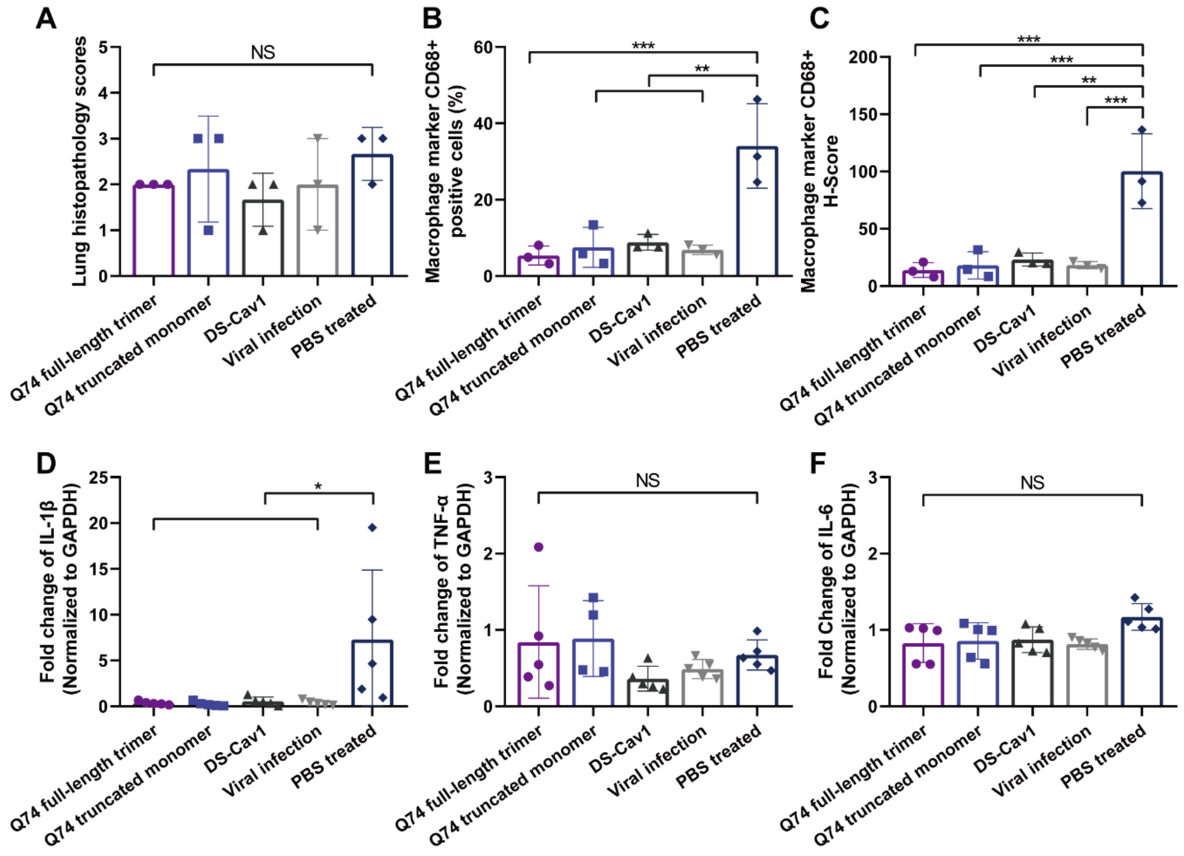

Figure S7. RSV infection induced lung inflammation, related to Figure 5. (A) Histopathological scores of RSV-induced lung tissue damage/inflammation. (B, C) Immunohistochemical analysis of $CD68^+$ macrophages: (B) Percentage of $CD68^+$ cells; (C) CD68 H-Scores. (D-F) Lung mRNA expression levels of proinflammatory cytokines: (D) IL-1β, (E) TNF-α, (F) IL-6, measured by RT-qPCR.

Table S1. Amino acid sequences of RSV F immunogens, related to Figure 1.

Design number	Sequence
Q74 full-length trimer	MELLILKANAITTILTAVTFCFASGQNITEEFYQSTCSAVSKGYLSAL<br>RTGWYTSVITIELSNIKENKCNGCDAKCKLIKQELDKYLNAVTELQ<br>LLMQSTPATNNGGSGGSGGSAIASGVAVSKVLHLEGEVNKIKSALLST<br>NKAVVSLSNGVSVLTSKVLDLKNYIDKQLLPIVNKQSCSISNIETVIEF<br>QQKNNRLLEITREFSVNAGVTTPVSTYMLTNSELLSLINDMPITNDQ<br>KKLMSNNVQIVRQQSYSIMSIIKEEVLAYVVQLPLYGVIDTPCWKLH<br>TSPLCTTNTKEGSNICLTRTDRGWYCDNAGSVSFFPQAETCKVQSNR<br>VFCDTMNSLTLPSEVNLCNVDIFNPKYDCKIMTSCTDVSSSVITSLGA<br>IVSCYGKTKCTASNKNRGIIKTFSNGCDYVSNKGVDTVSVGNTLYYV<br>NKQEGKSLYVKGEPIINFYDPLVFPCDEFDASISQVNEKINQSLAFIR<br>KSDELLSAIGGYIPEAPRDGQAYVRKDGEWVLLSTFL*
Q74 truncated monomer	MELLILKANAITTILTAVTFCFASGQNITEEFYQSTCSAVSKGYLSAL<br>RTGWYTSVITIELSNIKENKCNGCDAKCKLIKQELDKYLNAVTELQ<br>LLMQSTPATNNGGSGGSGGSAIASGVAVSKVLHLEGEVNKIKSALLST<br>NKAVVSLSNGVSVLTSKVLDLKNYIDKQLLPIVNKQSCSISNIETVIEF<br>QQKNNRLLEITREFSVNAGVTTPVSTYMLTNSELLSLINDMPITNDQ<br>KKLMSNNVQIVRQQSYSIMSIIKEEVLAYVVQLPLYGVIDTPCWKLH<br>TSPLCTTNTKEGSNICLTR*
DS-Cav1	MELLILKANAITTILTAVTFCFASGQNITEEFYQSTCSAVSKGYLSAL<br>RTGWYTSVITIELSNIKENKCNGTDAKVKLIKQELDKYKNAVTELQ<br>LLMQSTPATNNRARRELPRFMNYTLNNAKKTNVTLSKKRKRRFLG<br>FLLGVGSAIASGVAVCKVLHLEGEVNKIKSALLSTNKAVVSLSNGVSV<br>LTFKVLDLKNYIDKQLLPILNKQSCSISNIETVIEFQQKNNRLLEITR<br>EFSVNAGVTTPVSTYMLTNSELLSLINDMPITNDQKKLMSNNVQIVR<br>QQSYSIMCIIKEEVLAYVVQLPLYGVIDTPCWKLHTSPLCTTNTKEG<br>SNICLTRTDRGWYCDNAGSVSFFPQAETCKVQSNRVFCDTMNSLTL<br>PSEVNLCNVDIFNPKYDCKIMTSKTDVSSSVITSLGAIVSCYGKTKCT<br>ASNKNRGIIKTFSNGCDYVSNKGVDTVSVGNTLYYVNKQEGKSLYVK<br>GEPIINFYDPLVFPSDEFDASISQVNEKINQSLAFIRKSDELLSAIGGYIP<br>EAPRDGQAYVRKDGEWVLLSTFL*

Underline, signal sequence; brown, F2 region; green, F1 region; purple, T4 fibritin-trimerization foldon; red with underlined, variant
